# Controlled radical fluorination of poly(meth)acrylic acids in aqueous solution

**DOI:** 10.1038/s41467-017-00376-z

**Published:** 2017-08-17

**Authors:** Yucheng Dong, Zhentao Wang, Chaozhong Li

**Affiliations:** 1 Key Laboratory of Organofluorine Chemistry and Collaborative Innovation Center of Chemistry for Life Sciences, Shanghai Institute of Organic Chemistry, Chinese Academy of Sciences, 345 Lingling Road, Shanghai, 200032 China; 2 School of Materials and Chemical Engineering, Ningbo University of Technology, No. 201 Fenghua Road, Ningbo, 315211 China

## Abstract

Fluorinated alkenes exhibit very poor reactivity in copolymerization with non-fluorinated polar monomers such as acrylates. Herein we describe a convenient method for the synthesis of poly(vinyl fluoride-co-acrylic acid) and poly(2-fluoropropene-co-methacrylic acid) copolymers. Thus, the silver-catalyzed decarboxylative radical fluorination of poly(acrylic acid) with Selectfluor in water at room temperature affords poly(vinyl fluoride-co-acrylic acid) copolymers in high yields with well-defined molecular weights and polydispersities. A linear correlation is observed between the extent of fluorination and the amount of Selectfluor, indicating that the copolymer of virtually any monomer ratio can be readily accessed by controlling the amount of Selectfluor. This controlled decarboxylative fluorination is extended to poly(methacrylic acid), leading to well-defined poly(2-fluoropropene-co-methacrylic acid) copolymers.

## Abstract (web summary)

Fluorinated alkenes have very poor reactivity in copolymerizations with non-fluorinated polar monomers. Here the authors show silver-catalyzed decarboxylative radical fluorination of poly(acrylic acid) with Selectfluor in water at room temperature to form poly(vinyl fluoride-co-acrylic acid) copolymers.

## Introduction

Fluorinated polymers exhibit high thermostability, good hydrophobicity and lipophobicity, and low refractive index and friction coefficient. They are of low surface energy and resistant to UV, ageing, and chemicals. These unique characteristics make fluoropolymers of immense applications in many areas ranging from aerospace to optics and microelectronics and even to our daily lives^1^. However, fluoroplastics also have various drawbacks. For example, they are often crystalline and difficult to cure, and have poor cohesive and adhesive properties. One of the solutions to fluorinated materials of better performance is the copolymerization of fluorinated monomers with non-fluorinated ones^2–5^. The partially fluorinated copolymers may display the advantageous complementary properties deriving from the non-fluorinated and the fluorinated moieties while suppressing most of the respective points of weakness. The copolymerization of fluorinated alkenes with non-fluorinated ones has led to various industrial products such as ETFE (ethylene–tetrafluoroethylene copolymer), ECTFE (ethylene–chlorotrifluoroethylene copolymer) and TFEP (tetrafluoroethylene– propene copolymer), and is of growing interest^6^. However, while fluorinated alkenes such as tetrafluoroethylene or vinyl fluoride copolymerize nicely with a number of non-polar monomers such as ethylene or vinyl chloride^2, 6^, they exhibit extremely poor reactivity in copolymerization with non-fluorinated polar monomers such as styrene, acrylonitrile or acrylates^2, 7^. For example, the reactivity ratios in the copolymerization of vinyl fluoride and methyl acrylate irradiated with γ-rays were measured to be 0.009 and 43^7^. Hence, it is extremely difficult to prepare the well-defined copolymers of the two monomers by free-radical copolymerization, not to mention the control of monomer ratios, molecular weights, and polydispersities.

Although direct fluorination of polymers can be an effective method for the preparation of new materials^8, 9^, they require the use of highly toxic fluorinating agents and harsh reaction conditions^8–15^, such as treatment with F_2_^8–10^, XeF_2_^8, 9^, SF_6_ under electrical discharge^11^, BF_3_∙Et_2_O^12^, SF_4_/HF^13^, fluorinated peroxides^14^, and HF electrochemically^15^. As a consequence, these methods suffer from modest yields and undesirable side reactions, including loss of pendant functionality, degradation of molecular weight, and cross-linking of polymers. More importantly, no example of post-polymerization fluorination has ever been reported for the synthesis of fluorinated copolymers with well-defined structures.

We recently reported that^16^, under the catalysis of AgNO_3_, aliphatic carboxylic acids underwent efficient decarboxylative fluorination^16–21^ with Selectfluor reagent^22–24^ (1-chloromethyl-4-fluoro-diazoniabicyclo[2,2,2]octane bis(tetrafluoroborate)) in aqueous solution. In this catalytic radical transformation^25^, Selectfluor acted as both the fluorine source and the oxidant. We envisioned that such a transformation might be extended to the fluorination of poly(acrylic acids). By control of the amount of Selectfluor, the polymer might be partially fluorinated. The resulting fluorinated polymers are, in fact, the vinyl fluoride–acrylic acid copolymers.

Herein a convenient entry to poly(vinyl fluoride-co-acrylate) and poly(2-fluoropropene-co-methacrylate) copolymers by controlled decarboxylative radical fluorination^26, 27^ of poly(meth)acrylic acids is described. With this method, not only the molecular weights and polydispersities of these fluorocopolymers can be well defined, but also their monomer ratios can be adjusted at ease.

## Results

### Decarboxylative fluorination of poly(acrylic acid)

Thus, poly(acrylic acid) (**1**) derived from the commercially available poly(acrylic acid, sodium salt) (average *M*_w_ ~ 8000, 45 wt% in H_2_O, Aldrich) was directly treated with Selectfluor and AgNO_3_ in water at room temperature. In a set of nine experiments, the amount of Selectfluor varied from 10 to 90 mol %, while the molar ratio of Selectfluor to AgNO_3_ was fixed at 5:1 in all cases. After the reactions were complete (12 h), saturated aqueous NaCl solution was added. The Ag(I) was thus separated and recovered as AgCl precipitate. The solution was acidified (to pH = 3) with dilute HCl and then dialyzed. After freeze-drying, the products poly(vinyl fluoride-co-acrylic acid) (**2a**–**2i**) were obtained in satisfactory yields (Table 1). This direct fluorination of polymers led nicely to the synthesis of structurally well-defined fluorocopolymers.

**Table 1:** Controlled decarboxylative fluorination of poly(acrylic acid) 1


Product	**2a**	**2b**	**2c**	**2d**	**2e**	**2f**	**2g**	**2h**	**2i**
*y*	10	20	30	40	50	60	70	80	90
Yield (%)^a^	83	82	81	79	79	78	77	76	74
*x* (mol %)^b^	6.8	16	25	33	41	48	54	59	66

^a^Isolated yield based on **1**

^b^Calculated from the equation *x* = 36 ÷ (12 + 19 × C/F) × 100%, where C/F refers to the C/F mass ratio determined by elemental analysis

**Fig. 1:**
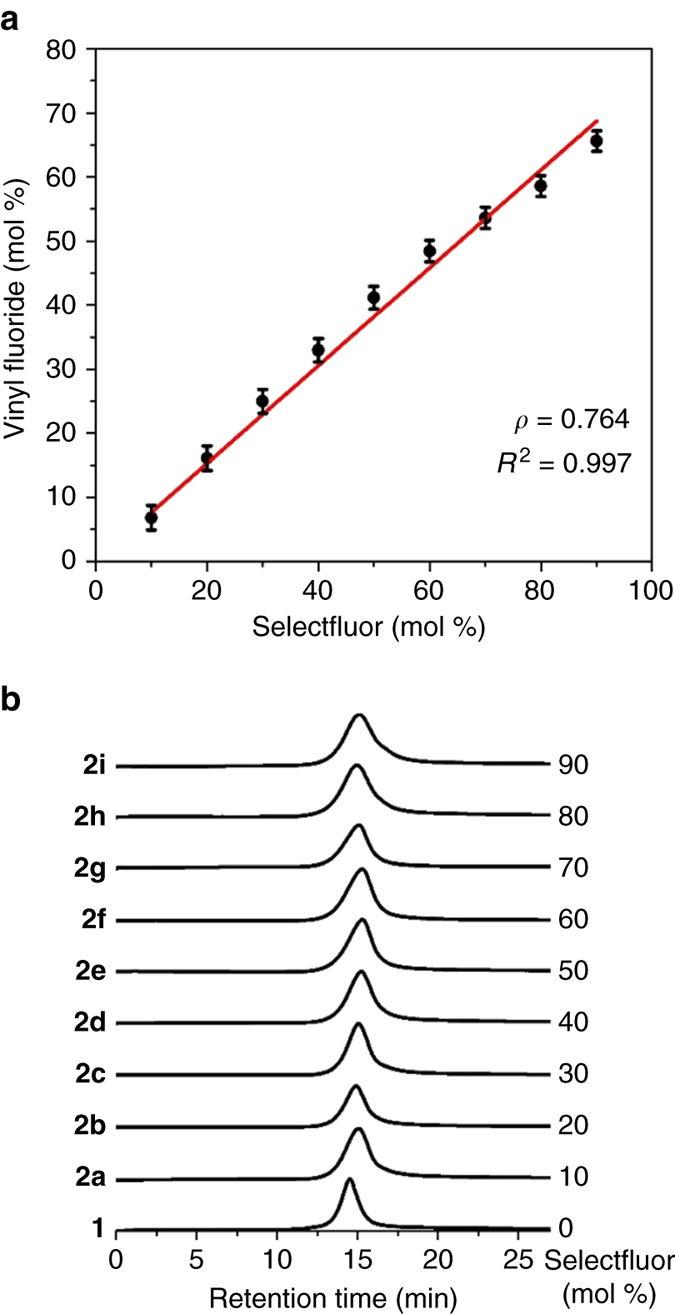
Controlled decarboxylative fluorination of poly(acrylic acid) 1 in water. **a** The dependence of the molar percentage of vinyl fluoride in copolymers **2** on the molar amount of Selectfluor. **b** GPC (DMF) chromatograms of copolymers **2a–2i** and polymer **1**

The fluorocopolymers **2a–2i** were then subjected to elemental analysis, based on which the C/F mass ratios were calculated. The extent of decarboxylative fluorination (x) was then calculated from the C/F mass ratio based on the equation *x* = 36 ÷ (12 + 19 × C/F), which was equivalent to the molar percentage of vinyl fluoride in copolymer **2** (see Supplementary Methods in the SI for details). The molar percentage of vinyl fluoride increased steadily from 6.8% in **2a** to 66% in **2i** when the amount of Selectfluor was increased from 10 mol % to 90 mol %. As a result, the solubility of **2** in water decreased from **2a** to **2i**. While **2a** and **2b** were soluble in water, **2c** was barely soluble, and **2d**–**2i** were insoluble. The solubility in *N*,*N*-dimethylformamide (DMF) also decreased from **2a** to **2i**, and **2 h** and **2i** showed poor solubility. The molar percentage of vinyl fluoride was then plotted against the amount of Selectfluor, as shown in Fig. 1a. A straight line could be drawn along those plots, indicating the linear dependence of the molar percentage of vinyl fluoride on the amount of Selectfluor. The monomer ratio of vinyl fluoride to acrylic acid in poly(vinyl fluoride-co-acrylic acid) **2** can thus be easily adjusted by the amount of Selectfluor, which in turn indicates that copolymer **2** of virtually any monomer ratio could be synthesized by this method.

The copolymers **2a–2i** thus obtained were fully characterized. Their gel permeation chromatography (GPC) analyses are summarized in Fig. 1b. The GPC chromatogram of **1** is also included in Fig. 1b for the ease of comparison. The GPC chromatograms of copolymers **2** showed close similarity to that of poly(acrylic acid) **1**. With the increase of the extent of fluorination, the polydispersity index (PDI) gradually increased from 1.38 in **1** to 1.61 in **2i** (Selectfluor = 90 mol %). These results indicate that the molecular weights and polydispersities of copolymers **2** are mainly determined by the starting material poly(acrylic acid) **1**, while fluorination lowers the molecular weight and slightly increases the PDI in a predictable pattern. The GPC data also imply that the decarboxylative fluorination of the polymer **1** takes place in a well-distributed manner. Differential scanning calorimetry experiments showed that copolymers **2a–2i** all had single glass transition temperatures (*T*_g_), suggesting that they are random copolymers. The *T*_g_ decreased steadily from 129 °C for **2a** to 81 °C for **2i**, all lower than that of **1** (*T*_g_ = 144 °C) but higher than that of poly(vinyl fluoride) (*T*_g_ = 50 °C)^28^. Thermal gravimetric analysis indicated that the 10% weight loss of copolymer degradation occurred at 285 ~ 335 °C for **2a–2i** (335 °C for **1** and 267 °C for poly(vinyl fluoride)) ^29^.

To test the generality of the above method, another sample of poly(acrylic acid, sodium salt) with a higher molecular weight (average *M*_w_ ~ 15,000, 35 wt% in H_2_O, Aldrich) was subjected to the same set of experiments with variable amounts of Selectfluor in water under the catalysis of AgNO_3_. The corresponding poly(vinyl fluoride-co-acrylic acid) copolymers were achieved in high yields (see SI for details). The linear dependence of the molar percentage of vinyl fluoride on the amount of Selectfluor was again observed, which was almost identical to that in Fig. 1a (see Supplementary Fig. 1). These results have clearly demonstrated the applicability of controlled decarboxylative fluorination in synthesizing vinyl fluoride–acrylic acid copolymers of different molecular weights.

### Decarboxylative fluorination of poly(methacrylic acid)

**Fig. 2:**
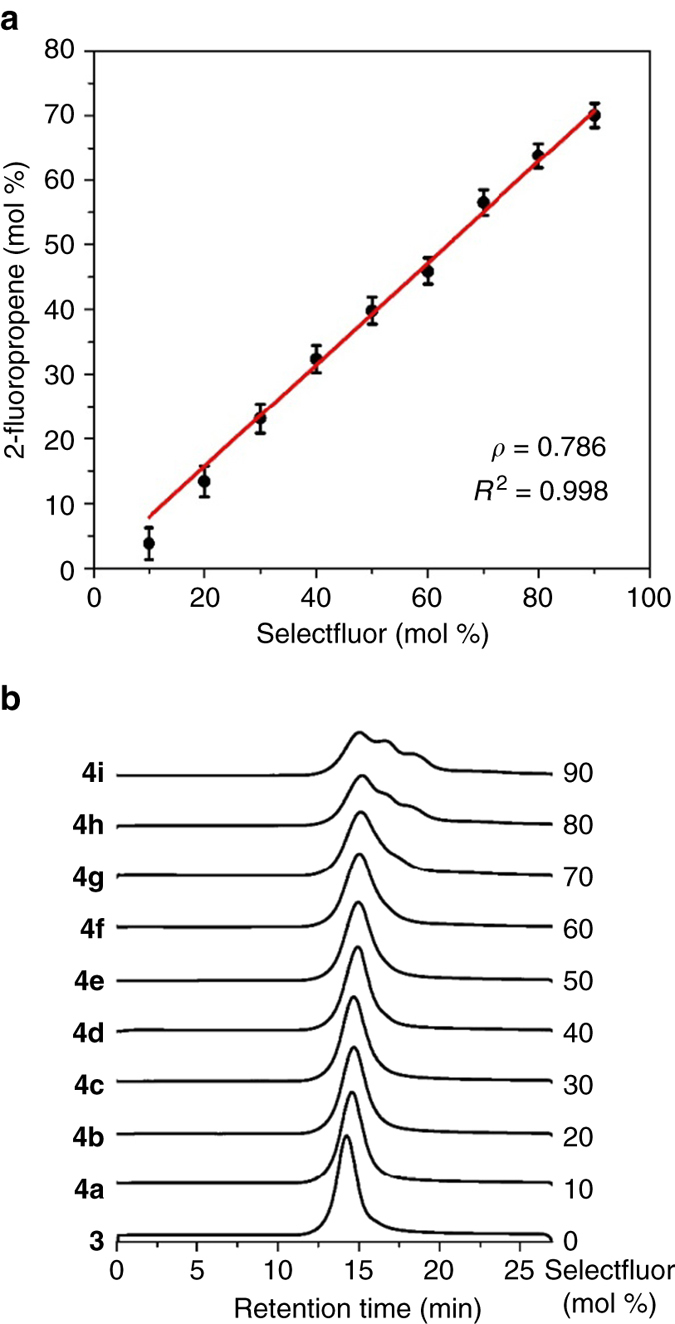
Controlled decarboxylative fluorination of PMAA **3** in water. **a** Dependence of the molar percentage of 2-fluoropropene in copolymers **4** on the amount of Selectfluor. **b** GPC (DMF) chromatograms of copolymers **4a–4i** and PMAA **3**

We then extended the above strategy to poly(methacrylic acid). Controlled decarboxylative fluorination of poly(methacrylic acid)^3^ derived from the commercially available poly(methacrylic acid, sodium salt) (Typical *M*_w_ 9500, 30 wt% in H_2_O, Aldrich) was carried out in water at room temperature, leading to the formation of 2-fluoropropene–methacrylic acid copolymers **4a–4i** in excellent yields (Table 2). By increasing the amount of Selectfluor from 10 to 90 mol % in a set of nine experiments, the molar content of 2-fluoropropene in **4** increased accordingly from 4% in **4a** to 70% in **4i**. Once again, an excellent linear correlation was observed between the amount of Selectfluor and the molar content of 2-fluoropropene monomer in copolymer **4**, as shown in Fig. 2a. The GPC chromatograms of **4a–4i** and **3** are also grouped in Fig. 2b. The PDI increased steadily from 1.45 for **4a** to 1.89 for **4g**. In the cases of **4h** and **4i**, significant broadening and multiple-peak GPC distribution were observed. This phenomenon might be attributed to the poor solubility of **4h** and **4i** in DMF at room temperature^30^.

**Table 2:** Controlled decarboxylative fluorination of poly(methacrylic acid) 3


Product	**4a**	**4b**	**4c**	**4d**	**4e**	**4f**	**4g**	**4h**	**4i**
*y*	10	20	30	40	50	60	70	80	90
Yield (%)^a^	81	76	74	80	74	91	87	83	93
*x* (mol %)^b^	3.8	13	23	32	40	46	56	64	70

^a^Isolated yield based on **3**

^b^Calculated from the equation *x* = 48 ÷ (12 + 19 × C/F) × 100%, where C/F refers to the C/F mass ratio determined by elemental analysis

## Discussion

Copolymers **2** and **4** contain both hydrophilic carboxyl groups and hydrophobic fluorine atoms attached to the polymer backbone, which are structurally distinct from poly(fluoroalkyl acrylate) with side-chain fluorination^31–34^. This unique structure should lead to different properties and applications of these fluorocopolymers. It is also expected that further development of radical fluorination methods with fluoride ion as the fluorine source^35–39^ may allow these fluorocopolymers to be accessed more economically, thus rendering them of more practical value.

In conclusion, we have successfully demonstrated that the previously inaccessible poly(vinyl fluoride-co-acrylic acid) and poly(2-fluoropropene-co-methacrylic acid) can be conveniently and efficiently accessed by silver-catalyzed decarboxylative fluorination of readily available poly(acrylic acid) and poly(methacrylic acid), respectively. This catalytic radical fluorination allows the site-specific introduction of fluorine atoms into polymers without the rupture of polymer chains. The operations are simple and the conditions are mild (room temperature, water as the solvent). The silver(I) ions can be easily removed and recovered as AgCl precipitate. More importantly, this method is advantageous in that the monomer ratios of copolymers can be easily controlled by the amount of Selectfluor reagent employed. The linear correlation between fluoroalkene and Selectfluor shown in Figs. 1a, 2a (and also in Supplementary Fig. 1) indicates that copolymers of virtually any monomer ratio can be synthesized. Furthermore, the molecular weights and polydispersities are well defined by the starting poly(meth)acrylic acids.

The chemistry detailed above also indicates that acrylic acid and methacrylic acid serve as the equivalents to vinyl fluoride and 2-fluoropropene, respectively, in fluoropolymer synthesis. It is conceivable that, if an acrylic acid–acrylonitrile copolymer is subjected to the controlled decarboxylative fluorination, acrylic acid–vinyl fluoride–acrylonitrile tri-component copolymers can be obtained. Since (meth)acrylic acid copolymerizes readily with a number of functionalized alkenes such as styrene, acrylonitrile, and acrylates^40^, a class of functionalized, fluoro-containing copolymers that are difficult if not impossible to be accessed by radical copolymerization can be synthesized. It should also be noted that (meth)acrylic acids are among the ideal substrates for controlled radical polymerization^41–43^. Thus, the combination of controlled radical polymerization with controlled decarboxylative fluorination should provide endless possibility to well-defined fluoro-containing copolymers.

## Methods

### Poly(vinyl fluoride-co-acrylic acid) 2a. typical procedure

Selectfluor (354 mg, 1.0 mmol), deionized water (26 mL) and sodium polyacrylate (1.6 mL, 1.3 g/mL, *M*_w_ ~ 8000, 45 wt% in H_2_O, equivalent to 10 mmol acrylic acid monomer) were added successively into a three-necked flask at room temperature under nitrogen atmosphere. The mixture was stirred at room temperature for 10 min with the aid of a magnetic bar. Silver nitrate (0.2 mmol, 2.0 mL, 0.1 mol/L solution in water) was then added. The reaction mixture was kept from light and stirred at room temperature for 12 h. Saturated NaCl solution (10 mL) was added and the mixture was stirred for 30 min. The resulting mixture was centrifuged. The white precipitate was separated from the solution by filtration. The precipitate was added into acetone (10 mL) and the mixture was again centrifuged. After filtration, the white precipitate was collected and dried under vacuum. AgCl was thus obtained as a white solid. Yield: 23 mg (80% based on AgNO_3_). The acetone solution and the aqueous solution were combined and then acidified with dilute hydrochloric acid until the pH was close to 3. The resulting solution was poured into a dialysis bag and dialyzed for 2 days. The solution was then freeze-dried. The product copolymer **2a** was thus obtained as a white powder. Yield: 583 mg (83%).

### Data availability

Supplementary information contains Supplementary Methods, Supplementary Tables 1 and 2, and Supplementary Figures 1–16. These data are available from the corresponding author (C.L.) upon reasonable request. (clig@mail.sioc.ac.cn).

## Electronic supplementary material

Supplementary Information

## References

[1] Ebnesajjad, S. (Ed.) *Introduction to Fluoropolymers: Materials, Technology, and Applications* (Elsevier, 2013).

[2] Ameduri, B. & Boutevin, B. *Well-Architectured Fluoropolymers: Synthesis, Properties and Applications* 187–230 (Elsevier, 2004).

[3] Boutevin B, Ameduri B (1994). Copolymerization of fluorinated monomers with nonfluorinated monomers. Reactivity and mechanisms. Macromol. Symp..

[4] Hougham, G., Cassidy, P., Johns, K. & Davidson, T. *Fluoropolymers* (Kluvert, 1999).

[5] Ameduri B (2009). From vinylidene fluoride (VDF) to the applications of VDF-containing polymers and copolymers: recent developments and future trends. Chem. Rev..

[6] Gangal, S. V. & Brothers, P. D. in *Encyclopedia of Polymer Science and Technology* 4th edn Vol. 9, 526–542 (ed. Mark, H. F.) (Wiley, 2014).

[7] Usmanov KhU, Sirlibaev TS, Yul’chibaev AA (1977). Vinyl fluoride polymers. Russ. Chem. Rev..

[8] Reisinger JJ, Hillmyer MA (2002). Synthesis of fluorinated polymers by chemical modification. Prog. Polym. Sci..

[9] Kharitonov, A. P. *Direct Fluorination of Polymers* (Nova Science, 2008).

[10] Dubois M (2005). Direct fluorination of poly(*p*-phenylene). Polymer.

[11] Das PS, Adhikari B, Maiti S (1994). Fluorination of polymers by sulfur hexafluoride gas under electric discharge. J. Polym. Sci. A Polym. Chem.

[12] Lienhard M (1997). Synthesis and characterization of the new fluoropolymer poly(difluorosilylenemethylene); an analogue of poly(vinylidene fluoride). J. Am. Chem. Soc..

[13] Nuyken O, Dannhorn W, Obrecht W (1994). Fluorination of polymers containing COOH/COOR-groups with SF_4_/HF. Marcomol. Chem. Phys..

[14] Zhou Z-B, He H-Y, Weng Z-Y, Qu Y-L, Zhao C-X (1996). Modification of polystyrene via aromatic per(poly)fluoroalkylation by per(poly)fluorodiacyl peroxides. J. Fluorine Chem..

[15] Noel M, Suryanarayanan V, Chellammal S (1997). A review of recent developments in the selective electrochemical fluorination of organic compounds. J. Fluorine Chem..

[16] Yin F, Wang Z, Li Z, Li C (2012). Silver-catalyzed decarboxylative fluorination of aliphatic carboxylic acids in aqueous solution. J. Am. Chem. Soc..

[17] Leung JCT (2012). Photo-fluorodecarboxylation of 2-aryloxy and 2-aryl carboxylic acids. Angew. Chem. Int. Ed..

[18] Rueda-Becerril M (2014). Direct C-F bond formation using photoredox catalysis. J. Am. Chem. Soc..

[19] Wu X (2015). Transition-metal-free visible-light photoredox catalysis at room-temperature for decarboxylative fluorination of aliphatic carboxylic acids by organic dyes. Chem. Commun..

[20] Ventre S, Petronijevic FR, MacMillan DWC (2015). Decarboxylative fluorination of aliphatic carboxylic acids via photoredox catalysis. J. Am. Chem. Soc..

[21] Zhang Q-W (2016). Fluorodecarboxylation for the synthesis of trifluoromethyl aryl ethers. Angew. Chem. Int. Ed..

[22] Banks RE, Mohialdin-Khaffaf SN, Lal GS, Sharif IRG (1992). Syvret, 1-Alkyl-4-fluoro-1,4-diazoniabicyclo[2.2.2]octane salts: a novel family of electrophilic fluorinating agents. J. Chem. Soc. Chem. Commun..

[23] Singh RP, Shreeve JM (2004). Recent highlights in electrophilic fluorination with 1-chloromethyl-4-fluoro-1,4-diazoniabicyclo[2.2.2]octane bis(tetrafluoroborate). Acc. Chem. Res..

[24] Nyffeler PT, Duron SG, Burkart MD, Vincent SP, Wong C-H (2005). electfluor: mechanistic insight and applications. Angew. Chem. Int. Ed..

[25] Studer A, Curran DP (2016). Catalysis of radical reactions: a radical chemistry perspective. Angew. Chem. Int. Ed..

[26] Chatalova-Sazepin C, Hemelaere R, Paquin J-F, Sammis GM (2015). Recent advances in radical fluorination. Synthesis.

[27] Sibi MP, Landais Y (2013). C_sp_3-F bond formation: a free-radical approach. Angew. Chem. Int. Ed..

[28] Hanes MD, Lando JB (1993). Thermal analysis of poly(vinyl fluoride). J. Appl. Polym. Sci..

[29] Raucher D, Levy M (1979). Thermal stability of homo- and copolymers of vinyl fluoride. J. Polym. Sci. Polym. Chem. Ed.

[30] Siebourg W, Lundberg RD, Lenz RW (1980). Gel permeation chromatographic characterization of sulfonated polystyrenes. Macromolecules.

[31] Hansen NML, Jankova K, Hvilsted S (2007). Fluoropolymer materials and architectures prepared by controlled radical polymerizations. Eur. Polym J..

[32] Hirao A, Sugiyama K, Yokoyama H (2007). Precise synthesis and surface structures of architectural per- and semifluorinated polymers with well-defined structures. Prog. Polym. Sci..

[33] Ameduri B (2010). Controlled radical (co)polymerization of fluoromonomers. Macromolecules.

[34] Yao W, Li Y, Huang X (2014). Fluorinated poly(meth)acrylate: synthesis and properties. Polymers.

[35] Liu W (2012). Oxidative aliphatic C-H fluorination with fluoride ion catalyzed by a manganese porphyrin. Science.

[36] Liu W, Groves JT (2013). Manganses-catalyzed oxidative benzylic C-H flurination by fluoride ions. Angew. Chem. Int. Ed..

[37] Liu W, Huang X, Groves JT (2013). Oxidative aliphatic C-H fluorination with manganese catalysts and fluoride ion. Nat. Protoc..

[38] Huang X (2014). Late stage benzylic C-H fluorination with [^18^F]fluoride for PET imaging. J. Am. Chem. Soc..

[39] Huang X, Liu W, Hooker JM, Groves JT (2015). Targeted fluorination with the fluoride ion by manganese-catalyzed decarboxylation. Angew. Chem. Int. Ed..

[40] Young LJ (1961). Copolymerization parameters. J. Polymer Sci.

[41] *Fundamentals of Controlled/Living Radical Polymerization* (eds Tsarevsky, N. V., Sumerlin, B. S.) (RSC, 2013).

[42] Matyjaszewski K, Tsarevsky NV (2014). Macromolecular engineering by atom transfer radical polymerization. J. Am. Chem. Soc..

[43] Braunecker WA, Matyjaszewski K (2007). Controlled/living radical polymerization: features, developments, and perspectives. Prog. Polym. Sci..

