# Supplementary material for: Controlled radical fluorination of poly(meth)acrylic acids in aqueous solution
Source: Nat Commun. 2017 Aug 17;8:277. doi: 10.1038/s41467-017-00376-z (PMC5561055; doi:10.1038/s41467-017-00376-z)
Supplement: Supplementary file 1 — Supplementary Information [file 41467_2017_376_MOESM1_ESM.pdf]

Description of Supplementary Files

File Name: Supplementary Information
Description: Supplementary Figures, Supplementary Tables and Supplementary Methods

Supplementary Methods

Reagents and materials

Poly(acrylic acid, sodium salt) (average M_w ~ 8000, 45 wt% in H_2O, Aldrich), poly(acrylic acid, sodium salt) (average M_w ~ 15000, 35 wt% in H_2O, Aldrich) and poly(methacrylic acid, sodium salt) (Typical M_n 5400, Typical M_w 9500, 30 wt% in H_2O, Aldrich) were purchased and directly used.

Silver nitrate (99.8% purity), Selectfluor (≥ 98.0% purity), *N*-methyl-*N*-nitroso-toluene-4-sulfonamide (98% purity), $(CF_3)_2CHOH$ and $PhCF_3$ were purchased and directly used. Solvents such as acetone, diethyl ether and *N*, *N*-dimethylformamide were distilled prior to use.

Regenerated cellulose dialysis membranes (M_wCO 2000; flat width, 45 mm; vol/length 6.4 mL/cm; diameter, 29 mm) were purchased from Shanghai Yuanye Biotechnology Co. Ltd. Microscope slides were from Sail Brand. Aluminum sheets (1.5 mm•25 mm•250 mm) and aluminum foils (0.03 mm•25 mm•500 mm) were from Huaquan Metal, Shanghai.

Measurements

^{1}H NMR spectra were recorded using a Bruker Avance III 400 spectrometer with dimethyl sulfoxide-d_6, acetone-d_6 or $CDCl_3$. ^{19}F NMR spectra were recorded using a Varian Mercury 300 spectrometer with H_2O or DMF. ^{13}C NMR spectra were recorded using an Agilent Technologies 400 MR spectrometer with D_2O or acetone-d_6 as the solvent.

Fourier transform infrared (FTIR) spectra were measured with a Thermo Nicolet iS5 spectrometer by KBr method. Polymer samples were dissolved in acetone and ground in KBr pellet before drying and tableting.

C, H, N elemental analysis (EA) was performed with an Elementar Vario MicroCube analyzer and F element analysis was conducted with an Elementar Vario EL III analyzer.

Gel permeation chromatography (GPC) was performed using a GPC Waters system equipped with a Waters 515 HPLC pump and a Waters 2414 refractive index detector. The GPC eluent was DMF or THF at a flow rate of 1.0 mL·min^{-1}. Calibration was performed with PMMA standards. Waters E2695 was for H_2O, and calibration was performed with PEG standards.

Differential scanning calorimetry (DSC) was performed with a TA Instruments Q10P calorimeter under nitrogen atmosphere at a heating rate of 10 °C·min^{-1}. The glass transition temperatures (T_g) were obtained from the second heating run.

Thermal gravimetric analysis (TGA) was performed with a TA Instruments Q500 analyzer at a heating rate of 10 °C·min^{-1} from room temperature to 600 °C under nitrogen atmosphere.

Methods of Calculations of Molar Percentage and Yield.

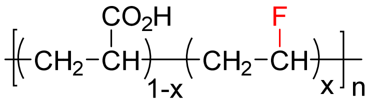

2a - 2i

The C/F mass ratio of copolymer **2** can be calculated as:

$$C/F = 12 \times (3\text{-}x) \div (19x) \qquad \text{(Eq 1)}$$

Thus, the molar percentage of vinyl fluoride in **2** can be deduced as:

$$x = 36 \div (12 + 19 \times C/F) \times 100\% \qquad \text{(Eq 2)}$$

In our reactions, polyacrylic acid **1** equivalent to 10 mmol of acrylic acid monomer was used. The theoretical yield of copolymer **2** can be calculated as 10 × (72 – 26x) mg. If the amount of **2** isolated experimentally is A mg, the isolated yield of **2** can then be calculated as

$$\text{Yield} = A \div (720 - 260x) \times 100\% \qquad \text{(Eq 3)}$$

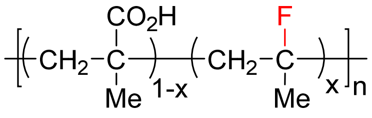

4a - 4i

For copolymer **4**, the C/F mass ratio can be calculated as:

$$C/F = 12 \times (4 - x) \div (19x) \qquad \text{(Eq 4)}$$

Thus, the molar percentage of 2-fluoropropene in **4** can be deduced as:

$$x = 48 \div (12 + 19 \times C/F) \times 100\% \qquad \text{(Eq 5)}$$

Again in our reactions, polymethacrylic acid **3** equivalent to 10 mmol of methacrylic acid monomer was used. The theoretical yield of copolymer **4** can be calculated as 10 × (86 – 26x) mg. If the amount of **4** isolated experimentally is A mg, the isolated yield of **4** can then be calculated as

$$\text{Yield} = A \div (860 - 260x) \times 100\% \qquad \text{(Eq 6)}$$

Silver-Catalyzed Controlled Decarboxylative Fluorination.

Controlled Decarboxylative Fluorination of PAA **1**.

Poly(acrylic acid) 1. Poly(acrylic acid, sodium salt) (1.6 mL, M_w ~ 8000, 1.3 g/mL, 45 wt% in H_2O, equivalent to 10 mmol acrylic acid) was diluted by H_2O (20 mL) and acidized to pH = 3 ~ 4 with dilute hydrochloric acid. The resulting solution was dialyzed for 2 days and then freeze-dried. Polymer **1** was thus obtained as a white solid. Yield: 606 mg (84%). FT-IR (KBr): *v* (cm^{-1}) 3437, 3129, 2952, 2598, 1717, 1452, 1411, 1245, 1170, 1044, 795. ^1H NMR (400 MHz, DMSO-d_6): δ 12.23 (br s, COOH), 2.21 (br s, CH), 1.87-1.65, 1.64-1.23 (all br, CH_2). ^{13}C NMR (101 MHz, D_2O): δ 178.98, 177.39 (s, C=O), 41.47(s), 40.53-35.57 (m), 35.05-33.24 (m), 31.23 (s). EA: C, 44.55%; H, 5.98%. GPC (DMF, PMMA calibration): M_n: 470 kDa, M_w: 649 kDa, PDI: 1.38. TGA: 402 °C (50% weight loss). DSC: T_g: 144 °C. Acid **1** was converted to its methyl ester by reaction with CH_2N_2 according to the literature method (Literature: Moore, J. A.; Reed, D. E. *Org. Synth.* **1961**, *41*, 16–20), which was further analyzed by GPC: GPC (DMF, PMMA calibration): M_n: 12.9 kDa, M_w: 19.4 kDa, PDI: 1.50. GPC (THF, PMMA calibration): M_n: 6.5 kDa, M_w: 12.4 kDa, PDI: 1.89.

Vinyl fluoride – acrylic acid copolymer 2a. Typical procedure. Selectfluor (354 mg, 1.0 mmol), deionized water (26 mL) and sodium polyacrylate (1.6 mL, 1.3 g/mL, M_w ~ 8000, 45 wt% in H_2O, equivalent to 10 mmol acrylic acid monomer) were added successively into a three-necked flask at room temperature under nitrogen atmosphere. The mixture was stirred at room temperature for 10 min with the aid of a magnetic bar. Silver nitrate (0.2 mmol, 2.0 mL, 0.1 mol/L solution in water) was then added. The reaction mixture was kept from light and stirred at room temperature for 12 h. Saturated NaCl solution (10 mL) was added and the mixture was stirred for 30 min. The resulting mixture was centrifuged. The white precipitate was separated from the solution by filtration. The precipitate was added into acetone (10 mL) and the mixture was again centrifuged. After filtration, the white precipitate was collected and dried under vacumn. AgCl was thus obtained as a white solid. Yield: 23 mg (80% based on $AgNO_3$). The acetone solution and the aqueous solution were combined and then acidified with dilute hydrochloric acid until the pH was close to 3. The resulting solution was poured into a dialysis bag and dialyzed for 2 days. The solution was then freeze-dried. The product copolymer **2a** was thus obtained as a white powder. Yield: 583 mg (83%). FT-IR (KBr): v (cm^{-1}) 3431, 3125, 2958, 2622, 1715, 1453, 1410, 1239, 1174, 1043, 800. ^1H NMR (400 MHz, DMSO-d_6): δ 12.21 (br s, COOH), 4.71-4.33 (br, F-CH), 2.22 (br s, CH), 1.75-1.66, 1.64-1.23 (all br, CH_2). ^{19}F NMR (282 MHz, H_2O): δ -184.0 (s)，-185.4 (s). ^{13}C NMR (101 MHz, D_2O): δ 178.64, 177.25 (s, C=O), 90.99-90.10 (m, C-F), 41.45 (s), 40.63-35.91 (m), 34.83-33.25 (m), 31.05 (s). EA: C, 48.06%; H, 5.60%; F, 1.77%. GPC (DMF, PMMA calibration): M_n: 340 kDa, M_w: 484 kDa, PDI: 1.42. TGA: 408 °C (50% weight loss). DSC: T_g: 129 °C. Acid **2a** was converted to its methyl ester by reaction with CH_2N_2 according to the literature method, which was further analyzed by GPC: GPC (DMF, PMMA calibration): M_n: 12.4 kDa, M_w: 18.9 kDa, PDI: 1.52. GPC (THF, PMMA calibration): M_n: 4.8 kDa, M_w: 8.3 kDa, PDI: 1.72.

Vinyl fluoride – acrylic acid copolymer 2b. This copolymer was prepared according to the procedure outlined in the synthesis of **2a** except that the amounts of Selectfluor,

deionized water and aqueous $AgNO_3$ (0.1 M) solution were 2.0 mmol, 34 mL and 4.0 mL, respectively. Copolymer **2b** was thus obtained as a white solid. Yield: 557 mg (82%). FT-IR (KBr): *v* (cm^{-1}) 3455, 3164, 2952, 2646, 1719, 1451, 1409, 1241, 1175, 1038, 804. ^{1}H NMR (400 MHz, DMSO-d_6): δ 12.30 (br s, COOH), 4.75-4.33 (br, F-CH), 2.21(br s, CH), 1.77-1.61, 1.61-1.33 (all br, CH_2). ^{19}F NMR (282 MHz, H_2O): δ -182.4 (s), -183.9 (s), -185.5 (s). ^{13}C NMR (101 MHz, D_2O): δ 178.52, 177.17 (s, C=O), 91.17-90.25 (m, C-F), 41.47 (s), 40.59-36.54 (m), 34.98-33.36 (m), 31.02 (s). EA: C, 46.31%; H, 5.96%; F, 4.15%. GPC (DMF, PMMA calibration): M_n: 348 kDa, M_w: 511 kDa, PDI: 1.46. TGA: 401 °C (50% weight loss). DSC: T_g: 123 °C. Acid **2b** was converted to its methyl ester by reaction with CH_2N_2 according to the literature method, which was further analyzed by GPC: GPC (DMF, PMMA calibration): M_n: 11.7 kDa, M_w: 18.0 kDa, PDI: 1.53. GPC (THF, PMMA calibration): M_n: 4.6 kDa, M_w: 7.6 kDa, PDI: 1.66.

Vinyl fluoride – acrylic acid copolymer 2c. This copolymer was prepared according to the procedure outlined in the synthesis of **2a** except that the amounts of Selectfluor, deionized water and aqueous $AgNO_3$ (0.1 M) solution were 3.0 mmol, 42 mL and 6.0 mL, respectively. Copolymer **2c** was thus obtained as a white solid. Yield: 530 mg (81%). FT-IR (KBr): *v* (cm^{-1}) 3428, 2959, 2622, 1724, 1447, 1409, 1364, 1181, 1043, 816. ^{1}H NMR (400 MHz, DMSO-d_6): δ 12.32 (br s, COOH), 4.83-4.33 (br s, F-CH), 2.70-2.22 (all br, CH), 2.01-1.61, 1.58-1.34 (all br, CH_2). ^{19}F NMR (282 MHz, H_2O): δ -182.7 (s)，-183.9 (s)，-185.4 (s). ^{13}C NMR (101 MHz, D_2O/acetone-d_6): δ 179.59, 177.17, 175.51 (s, C=O), 91.37-87.68 (m, C-F), 41.97-36.57 (m), 35.97-33.72 (m), 31.01 (s). EA: C, 47.42%; H, 5.71%; F, 6.81%. GPC (DMF, PMMA calibration): M_n: 309 kDa, M_w: 461 kDa, PDI: 1.49. TGA: 407 °C (50% weight loss). DSC: T_g: 114 °C. Acid **2c** was converted to its methyl ester by reaction with CH_2N_2 according to the literature method, which was further analyzed by GPC: GPC (DMF, PMMA calibration): M_n: 11.5 kDa, M_w: 17.6 kDa, PDI: 1.52. GPC (THF, PMMA calibration): M_n: 4.9 kDa, M_w: 7.8 kDa, PDI: 1.57.

Vinyl fluoride – acrylic acid copolymer 2d. This copolymer was prepared according to the procedure outlined in the synthesis of **2a** except that the amounts of Selectfluor, deionized water and aqueous $AgNO_3$ (0.1 M) solution were 4.0 mmol, 70 mL and 8.0 mL, respectively. Copolymer **2d** was thus obtained as a white solid. Yield: 502 mg (79%). FT-IR (KBr): ν (cm^{-1}) 3428, 3123, 2961, 2640, 1716, 1445, 1413, 1366, 1235, 1180, 1043, 814. ^{1}H NMR (400 MHz, DMSO-d_6): δ 12.34 (br s, COOH), 4.96-4.35 (br s, F-CH), 2.76-2.21 (br, CH), 2.10-1.34 (br s, CH_2). ^{19}F NMR (282 MHz, DMF): δ -182.6 (s), -183.9 (s), -185.7 (s), -187.6 (s). ^{13}C NMR (101 MHz, acetone-d_6): δ 176.25, 174.20 (s, C=O), 91.35-87.87 (m, C-F), 41.85-36.55 (m), 36.08-33.67 (m), 31.04 (s). EA: C, 48.32%; H, 5.85%; F, 9.43%. GPC (DMF, PMMA calibration): M_n: 294 kDa, M_w: 443 kDa, PDI: 1.51. TGA: 415 °C (50% weight loss). DSC: T_g: 112 °C. Acid **2d** was converted to its methyl ester by reaction with CH_2N_2 according to the literature method, which was further analyzed by GPC: GPC (DMF, PMMA calibration): M_n: 11.8 kDa, M_w: 18.1 kDa, PDI: 1.53. GPC (THF, PMMA calibration): M_n: 4.6 kDa, M_w: 7.2 kDa, PDI: 1.56.

Vinyl fluoride – acrylic acid copolymer 2e. This copolymer was prepared according to the procedure outlined in the synthesis of **2a** except that the amounts of Selectfluor, deionized water and aqueous $AgNO_3$ (0.1 M) solution were 5.0 mmol, 88 mL and 10 mL, respectively. Copolymer **2e** was thus obtained as a white solid. Yield: 485 mg (79%). FT-IR (KBr): ν (cm^{-1}) 3419, 3119, 2964, 2634, 1712, 1443, 1409, 1366, 1236, 1179, 1044, 817. ^{1}H NMR (400 MHz, DMSO-d_6): δ 12.35 (br s, COOH), 4.98-4.34 (br, F-CH), 2.78-2.16 (br, CH), 2.11-1.33 (br s, CH_2). ^{19}F NMR (282 MHz, DMF): δ -184.9 (s), -186.4 (s), -188.0 (s), -190.0 (s). ^{13}C NMR (101 MHz, acetone-d_6): δ 176.08, 174.03 (s, C=O), 91.36-85.82 (m, C-F), 41.77-36.34 (m), 35.97-33.48 (m), 30.84 (s). EA: C, 47.47%; H, 5.93%; F, 11.94%. GPC (DMF, PMMA calibration): M_n: 307 kDa, M_w: 467 kDa, PDI: 1.52. TGA: 418 °C (50% weight loss). DSC: T_g: 106 °C. Acid **2e** was converted to its methyl ester by reaction with CH_2N_2 according to the literature method, which was further analyzed by GPC: GPC (DMF, PMMA calibration): M_n: 12.0 kDa, M_w: 18.7 kDa, PDI: 1.55. GPC (THF, PMMA calibration):

M_n: 4.2 kDa, M_w: 6.9 kDa, PDI: 1.65.

Vinyl fluoride – acrylic acid copolymer 2f. This copolymer was prepared according to the procedure outlined in the synthesis of **2a** except that the amounts of Selectfluor, deionized water and aqueous $AgNO_3$ (0.1 M) solution were 6.0 mmol, 86 mL and 12 mL, respectively. Copolymer **2f** was thus obtained as a white solid. Yield: 463 mg (78%). FT-IR (KBr): v (cm^{-1}) 3425, 3117, 2963, 2652, 1712, 1440, 1403, 1366, 1178, 1043, 818. ^1H NMR (400 MHz, DMSO-d_6): δ 12.36 (br s, COOH), 5.00-4.37 (br, CH), 2.78-2.17 (br, F-CH), 2.13-1.33 (br s, CH_2). ^{19}F NMR (282 MHz, DMF): δ -185.0 (s), -186.5 (s), -188.2 (s), -190.6 (s). ^{13}C NMR (101 MHz, acetone-d_6): δ 175.88, 173.67 (s, C=O), 90.23-85.64 (m, C-F), 41.35-36.73 (m), 35.36-33.83 (m), 30.84 (s). EA: C, 49.40%; H, 5.82%; F, 15.05%. GPC (DMF, PMMA calibration): M_n: 308 kDa, M_w: 472 kDa, PDI: 1.53. TGA: 415 °C (50% weight loss). DSC: T_g: 97 °C. Acid **2f** was converted to its methyl ester by reaction with CH_2N_2 according to the literature method, which was further analyzed by GPC: GPC (DMF, PMMA calibration): M_n: 11.7 kDa, M_w: 18.9 kDa, PDI: 1.61. GPC (THF, PMMA calibration): M_n: 4.0 kDa, M_w: 6.5 kDa, PDI: 1.62.

Vinyl fluoride – acrylic acid copolymer 2g. This copolymer was prepared according to the procedure outlined in the synthesis of **2a** except that the amounts of Selectfluor, deionized water and aqueous $AgNO_3$ (0.1 M) solution were 7.0 mmol, 84 mL and 14 mL, respectively. Copolymer **2g** was thus obtained as a white solid. Yield: 450 mg (77%). FT-IR (KBr): v (cm^{-1}) 3419, 3117, 2963, 2628, 1726, 1439, 1409, 1366, 1178, 1043, 818. ^1H NMR (400 MHz, DMSO-d_6): δ 12.40 (br, COOH), 5.01-4.43 (br, F-CH), 2.96-2.74, 2.68-2.19 (all br, CH), 2.15-1.34 (br s, CH_2). ^{19}F NMR (282 MHz, DMF): δ -186.8 (s), -188.3 (s), -189.9 (s), -192.5 (s). ^{13}C NMR (101 MHz, acetone-d_6): δ 175.88, 173.67 (s, C=O), 91.16-85.81 (m, C-F), 41.73-36.23 (m), 36.05-33.40 (m), 30.82 (s). EA: C, 48.93%; H, 6.19%; F, 16.84%. GPC (DMF, PMMA calibration): M_n: 335 kDa, M_w: 519 kDa, PDI: 1.55. TGA: 420 °C (50% weight loss). DSC: T_g: 91 °C. Acid **2g** was converted to its methyl ester by reaction with CH_2N_2 according to the

literature method, which was further analyzed by GPC: GPC (DMF, PMMA calibration): M_n: 13 kDa, M_w: 26.4 kDa, PDI: 2.03. The ester was not soluble in THF.

Vinyl fluoride – acrylic acid copolymer 2h. This copolymer was prepared according to the procedure outlined in the synthesis of **2a** except that the amounts of Selectfluor, deionized water and aqueous $AgNO_3$ (0.1 M) solution were 8.0 mmol, 82 mL and 16 mL, respectively. Copolymer **2h** was thus obtained as a yellowish solid. Yield: 433 mg (76%). FT-IR (KBr): v (cm^{-1}) 3425, 3114, 2959, 2628, 1712, 1436, 1408, 1367, 1178, 1122, 1042, 818. ^1H NMR (400 MHz, DMSO-d_6): δ 12.38 (br s, COOH), 5.02-4.43 (br, F-CH), 2.92-2.16 (all br, CH), 2.13-1.17 (br s, CH_2). ^{19}F NMR (282 MHz, DMF): δ -185.3 (s)，-186.8 (s, weak)，-188.3 (s)，-191.0 (s). ^{13}C NMR (101 MHz, acetone-d_6): δ 175.83, 173.64 (s, C=O), 91.16-85.88 (m, C-F), 42.16-36.42 (m), 36.10-33.44 (m), 30.87 (s). EA: C, 49.13%; H, 6.14%; F, 18.86%. GPC (DMF, PMMA calibration): M_n: 329 kDa, M_w: 520 kDa, PDI: 1.58. TGA: 421 °C (50% weight loss). DSC: T_g: 84 °C. Acid **2h** was converted to its methyl ester by reaction with CH_2N_2 according to the literature method, which was further analyzed by GPC: GPC (DMF, PMMA calibration): M_n: 11.7 kDa, M_w: 25.7 kDa, PDI: 2.19. The ester was not soluble in THF.

Vinyl fluoride – acrylic acid copolymer 2i. This copolymer was prepared according to the procedure outlined in the synthesis of **2a** except that the amounts of Selectfluor, deionized water and aqueous $AgNO_3$ (0.1 M) solution were 9.0 mmol, 80 mL and 18 mL, respectively. Copolymer **2i** was thus obtained as a yellowish solid. Yield: 404 mg (74%). FT-IR (KBr): v (cm^{-1}) 3431, 3117, 2960, 2634, 1713, 1436, 1409, 1367, 1178, 1122, 1042, 819. ^1H NMR (400 MHz, DMSO-d_6): δ 12.39 (br, COOH), 5.03-4.49 (br, F-CH), 2.93-2.17 (all br, CH), 2.16-1.14 (br s, CH_2). ^{19}F NMR (282 MHz, DMF): δ -185.2 (s),-186.6 (s, very weak), -188.2 (s), -191.0 (s). ^{13}C NMR (101 MHz, acetone-d_6): δ 176.25, 174.22 (s, C=O), 90.25-85.82 (m, C-F), 41.56-36.54 (m), 35.68-34.28 (m), 31.01 (s). EA: C, 49.27%; H, 5.99%; F, 21.82%. GPC (DMF, PMMA calibration): M_n: 286 kDa, M_w: 461 kDa, PDI: 1.61. TGA: 421 °C (50%

weight loss). DSC: T_g: 81 °C. Acid **2h** was converted to its methyl ester by reaction with CH_2N_2 according to the literature method, which was further analyzed by GPC: GPC (DMF, PMMA calibration): M_n: 17.1 kDa, M_w: 91.4 kDa, PDI: 5.33. The ester was not soluble in THF.

Controlled Decarboxylative Fluorination of Poly(acrylic acid) **1'**.

Poly(acrylic acid) 1'. Sodium polyacrylate water solution (2.15 mL, M_w ~ 15000, 1.25 g/mL, 35 wt% in H_2O, equivalent to 10 mmol acrylic acid monomer) was diluted by H_2O (20 mL) and acidized to pH = 3 ~ 4 with dilute hydrochloric acid. The resulting solution was dialyzed for 2 days and then freeze-dried. Polymer **1** was thus obtained as a white solid. Yield: 647 mg (90%). FT-IR (KBr): *v* (cm^{-1}) 3450, 3120, 2963, 2927, 2860, 2619, 1721, 1452, 1407, 1249, 1175, 1113, 915,798. ^{1}H NMR (400 MHz, DMSO-d_6): δ 12.24 (br s, COOH), 2.20 (br s, CH), 1.87-1.65, 1.64-1.25 (all br, CH_2). EA: C, 47.90%; H, 6.30%. The sodium polyacrylate purchased was directly used for GPC anaylsis: GPC (sodium salt of **1'**) (H_2O, PEG calibration): M_n: 446 kDa, M_w: 532 kDa, PDI: 1.19.

With the use of poly(acrylic acid, sodium salt) (M_w ~ 15000, d = 1.25 g/mL, 35 wt% in H_2O, Aldrich) as the starting material, the above procedures outlined in the synthesis of copolymers **2a – 2f** were followed to give copolymers **2a' – 2f'**. In GPC measurements, copolymers **2a' – 2f'** could not be easily eluted in DMF or THF. Thus, the copolymers were converted to their sodium salts by treatment with aqueous NaOH (0.1 N) solution, and the sodium salts were used for GPC analysis.

Vinyl fluoride – acrylic acid copolymer 2a'. Yield: 613 mg (87%). White solid. FT-IR (KBr): *v* (cm^{-1}) 3430, 3125, 2959, 2924, 2853, 2598, 1712, 1453, 1409, 1249, 1173, 1117, 800. ^{1}H NMR (400 MHz, DMSO-d_6): δ 12.28 (br s, COOH), 4.63-4.33 (br, F-CH), 2.22 (br s, CH), 1.89-1.68, 1.49-1.23 (all br, CH_2). ^{19}F NMR (282 MHz,

DMF-H_2O): δ -185.3, -186.7 (s), -188.5 (s), -190.3. EA: C, 45.08%; H, 6.04%; F, 1.75%. GPC (sodium salt of **2a'**) (H_2O, PEG calibration): M_n: 457 kDa, M_w: 561 kDa, PDI: 1.23.

Vinyl fluoride – acrylic acid copolymer 2b'. Yield: 577 mg (85%). White solid. FT-IR (KBr): *ν* (cm^{-1}) 3715, 3114, 2958, 2932, 2646, 1717, 1451, 1409, 1247, 1175, 1115, 805. ^1H NMR (400 MHz, DMSO-d_6): δ 12.30 (br s, COOH), 4.74-4.32 (br, F-CH), 2.63-2.37, 2.21 (br, CH), 1.81-1.63, 1.63-1.28 (all br, CH_2). ^{19}F NMR (282 MHz, DMF-H_2O): δ -185.3 (s), -186.5 (s), -188.4 (s), -190.1 (s). EA: C, 47.74%; H, 6.11%; F, 4.31%. GPC (sodium salt of **2b'**) (H_2O, PEG calibration): M_n: 491 kDa, M_w: 611 kDa, PDI: 1.25.

Vinyl fluoride – acrylic acid copolymer 2c'. Yield: 563 mg (86%). White solid. FT-IR (KBr): *ν* (cm^{-1}) 3441, 3152, 2963, 2930, 2622, 1712, 1450, 1411, 1364, 1250, 1178, 1124, 806. ^1H NMR (400 MHz, DMSO-d_6): δ 12.47 (br s, COOH), 4.86-4.31 (br s, F-CH), 2.71-2.36, 2.24 (all br, CH), 2.08-1.62, 1.60-1.30 (all br, CH_2). ^{19}F NMR (282 MHz, DMF): δ -185.3 (s), -186.5 (s), -188.3 (s), -190.0 (s). EA: C, 48.18%; H, 6.20%; F, 6.79%. GPC (sodium salt of **2c'**) (H_2O, PEG calibration): M_n: 476 kDa, M_w: 632 kDa, PDI: 1.33.

Vinyl fluoride – acrylic acid copolymer 2d'. Yield: 540 mg (85%). White solid. FT-IR (KBr): *ν* (cm^{-1}) 3458, 3169, 2963, 2933, 2860, 2627, 1712, 1447, 1409, 1364, 1254, 1182, 1118, 812. ^1H NMR (400 MHz, DMSO-d_6): δ 12.39 (br s, COOH), 4.96-4.31 (br s, F-CH), 2.68-2.14 (br, CH), 2.08-1.27 (br s, CH_2). ^{19}F NMR (282 MHz, DMF): δ -185.3 (s), -186.7 (s), -188.3 (s), -190.2 (s). EA: C, 49.86%; H, 6.39%; F, 9.70%. GPC (sodium salt of **2d'**) (H_2O, PEG calibration): M_n: 527 kDa, M_w: 706 kDa, PDI: 1.34.

Vinyl fluoride – acrylic acid copolymer 2e'. Yield: 507 mg (83%). White solid. FT-IR (KBr): *ν* (cm^{-1}) 3448, 3217, 2964, 2928, 2860, 2610, 1717, 1444, 1407, 1364,

1259, 1186, 1118, 812. ^{1}H NMR (400 MHz, DMSO-d_6): δ 12.40 (br s, COOH), 4.96-4.37 (br, F-CH), 2.67-2.17 (br, CH), 2.07-1.33 (br s, CH_2). ^{19}F NMR (282 MHz, DMF): δ -185.3 (s), -186.6 (s), -188.3 (s), -190.3 (s). EA: C, 49.78%; H, 6.32%; F, 12.40%. GPC (sodium salt of **2e'**) (H_2O, PEG calibration): M_n: 555 kDa, M_w: 744 kDa, PDI: 1.34.

Vinyl fluoride – acrylic acid copolymer 2f'. Yield: 491 mg (83%). White solid. FT-IR (KBr): *v* (cm^{-1}) 3449, 3117, 2967, 2929, 2623, 1713, 1442, 1408, 1366, 1256, 1183, 1117, 814. ^{1}H NMR (400 MHz, DMSO-d_6): δ 12.39 (br s, COOH), 4.97-4.36 (br, CH), 2.69-2.17 (br, F-CH), 2.11-1.35 (br s, CH_2). ^{19}F NMR (282 MHz, DMF): δ -185.3 (s), -186.6 (s), -188.3 (s), -190.7 (s). EA: C, 49.79%; H, 6.28%; F, 15.13%. GPC (sodium salt of **2f'**) (H_2O, PEG calibration): M_n: 665 kDa, M_w: 844 kDa, PDI: 1.27.

Controlled Decarboxylative Fluorination of PMAA **3**.

Poly(methacrylic acid) 3. Poly(methacrylic acid, sodium salt) water solution (2.9 mL, M_n ~ 5400, M_w ~ 9500, 1.251 g/mL, 30 wt% in H_2O, equivalent to 10 mmol methacrylic acid monomer) was added diluted in 20 mL H_2O and acidized to pH = 3 ~ 4 with dilute hydrochloric acid. The resulting solution was dialyzed for 2 days and then freeze-dried. Polymer **3** was thus obtained as a white solid. Yield: 703 mg (82%). FT-IR (KBr): *v* (cm^{-1}) 3437, 3179, 2998, 2946, 2598, 1705, 1485, 1450, 1389, 1263, 1176, 964, 930, 829. ^{1}H NMR (400 MHz, DMSO-d_6): δ 12.34 (br s, COOH), 2.16-1.54 (br, CH_2), 1.44-1.30, 1.28-1.13, 1.02, 0.93 (br s, CH_3). ^{13}C NMR (101 MHz, DMSO-d_6): δ 180.24, 179.98, 179.50, 179.32 (s, C=O), 54.90-53.38, 52.19-50.10 (m, HOOC-C), 44.92, 44.52 (s, CH_2), 18.92-17.93, 17.29-16.02 (m, CH_3). GPC (DMF, PMMA calibration): M_n: 288 kDa, M_w: 368 kDa, PDI: 1.28. TGA: 428 °C (50% weight loss). DSC: T_g: 161 °C.

2-Fluoropropene – methacrylic acid copolymer 4a. Typical Procedure. Selectfluor

(354 mg, 1.0 mmol), deionized water (16 mL) and sodium polymethacrylate (2.9 mL, M_n ~ 5400, M_w ~ 9500, 1.251 g/mL, 30 wt% in H_2O, equivalent to 10 mmol methacrylic acid monomer) were added successively into a three-necked flask at room temperature under nitrogen atmosphere. The mixture was stirred at room temperature for 10 min with the aid of a magnetic bar. Silver nitrate (0.2 mmol, 2.0 mL, 0.1 mol/L solution in water) was then added. The reaction mixture was kept from light and stirred at room temperature for 12 h. Saturated NaCl solution (10 mL) was added and the mixture was stirred for 30 min. The resulting mixture was centrifuged. The white precipitate was separated from the solution by filtration. The precipitate was added into acetone (10 mL) and the mixture was again centrifuged. After filtration, the white precipitate was collected and dried under vacumn. AgCl was thus obtained as a white solid. Yield: 24 mg (85% based on $AgNO_3$). The acetone solution and the aqueous solution were combined and then acidified with dilute hydrochloric acid until the pH was close to 3. The resulting solution was poured into a dialysis bag and dialyzed for 2 days. The solution was then freeze-dried. The product copolymer **4a** was thus obtained as a white solid. Yield: 687 mg (81%). FT-IR (KBr): ν (cm^{-1}) 3452, 3179, 2996, 2945, 2600, 1701, 1484, 1452, 1389, 1263, 1176, 964, 936, 829. ^1H NMR (400 MHz, DMSO-d_6): δ 12.34 (br s, COOH), 2.08-1.55 (br, CH_2), 1.45-1.29, 1.23-1.12 (br, CF-CH_3), 1.02, 0.93 (br s, HOOCC-CH_3). ^{19}F NMR (282 MHz, DMSO-d_6): δ -135.6 ~ -138.0 (br), -139.3 (s). ^{13}C NMR (101 MHz, DMSO-d_6) δ 180.23, 179.98, 179.50, 179.30 (s, C=O), 54.94-53.32, 52.52-49.66 (m, HOOC-C), 44.92, 44.52 (s, CH_2), 18.97-18.01, 17.11-16.16 (m, CH_3). EA: C, 50.74%; H, 7.37%; F, 0.77%. GPC (DMF, PMMA calibration): M_n: 264 kDa, M_w: 384 kDa, PDI: 1.45. TGA: 403 °C (50% weight loss). DSC: T_g: 161 °C.

2-Fluoropropene – methacrylic acid copolymer 4b. This copolymer was prepared according to the procedure outlined in the synthesis of **4a** except that the amounts of Selectfluor, deionized water and aqueous $AgNO_3$ (0.1 M) solution were 2.0 mmol, 33 mL and 4.0 mL, respectively. Copolymer **4b** was thus obtained as a white solid. Yield: 631 mg (76%). FT-IR (KBr): ν (cm^{-1}) 3437, 3173, 2995, 2946, 2600, 1705, 1481,

1455, 1388, 1262, 1178, 963, 933, 822. ^{1}H NMR (400 MHz, DMSO-d_6): δ 12.36 (br s, COOH), 2.19-1.56 (br, CH_2), 1.56-1.33, 1.27-1.13 (br, CF-CH_3), 1.03, 0.93 (br s, HOOCC-CH_3). ^{19}F NMR (282 MHz, DMSO-d_6): δ -135.8 ~ -138.3 (br), -138.5 ~ -140.0 (s). ^{13}C NMR (101 MHz, DMSO-d_6): δ 180.25, 179.98, 179.46, 179.29 (s, C=O), 98.66-96.32 (m, F-C), 54.94-53.45, 52.32-50.23 (m, HOOC-C), 44.93, 44.53 (s, CH_2), 18.90-18.04, 17.13- 16.17 (m, CH_3). EA: C, 51.88%; H, 7.51%; F, 2.84%. GPC (DMF, PMMA calibration): M_n: 240 kDa, M_w: 359 kDa, PDI: 1.49. TGA: 399 °C (50% weight loss). DSC: T_g: 158 °C.

2-Fluoropropene – methacrylic acid copolymer 4c. This copolymer was prepared according to the procedure outlined in the synthesis of **4a** except that the amounts of Selectfluor, deionized water and aqueous $AgNO_3$ (0.1 M) solution were 3.0 mmol, 34 mL and 6.0 mL, respectively. Copolymer **4c** was thus obtained as a white solid. Yield: 590 mg (74%). FT-IR (KBr): *v* (cm^{-1}) 3443, 3185, 2995, 2946, 2610, 1700, 1481, 1450, 1389, 1263, 1178, 963, 936, 833. ^{1}H NMR (400 MHz, DMSO-d_6): δ 12.38 (br s, COOH), 2.24-1.57 (br, CH_2), 1.57-1.33, 1.33-1.20 (br, CF-CH_3), 1.04, 0.94 (br s, HOOCC-CH_3). ^{19}F NMR (282 MHz, DMSO-d_6): δ -139.5 ~ -142.5 (br), -143.4 (s). ^{13}C NMR (101 MHz, DMSO-d_6): δ 180.54-178.66 (s, C=O), 99.09-96.27 (m, F-C), 54.83-49.28 (m, HOOC-C), 45.52-43.46 (m, CH_2), 27.36-20.18, 19.62-17.87, 17.28-16.11 (m, CH_3). EA: C, 52.07%; H, 7.57%; F, 5.05%. GPC (DMF, PMMA calibration): M_n: 234000, M_w: 355000, PDI: 1.52. TGA: 387 °C (50% weight loss). DSC: T_g: 148 °C.

2-Fluoropropene – methacrylic acid copolymer 4d. Typical Procedure. This copolymer was prepared according to the procedure outlined in the synthesis of **4a** except that the amounts of Selectfluor, deionized water and aqueous $AgNO_3$ (0.1 M) solution were 4.0 mmol, 52 mL and 8.0 mL, respectively. Yield: 620 mg (80%). FT-IR (KBr): *v* (cm^{-1}) 3431, 3161, 2993, 2942, 2616, 1704, 1478, 1451, 1388, 1260, 1182, 943, 834. ^{1}H NMR (400 MHz, DMSO-d_6): δ 12.41 (br s, COOH), 2.34-1.85, 1.85-1.57 (br, CH_2), 1.57-1.34, 1.34-1.19 (br, CF-CH_3), 1.19-1.00, 1.00-0.77 (br,

HOOCC-CH_3). ^{19}F NMR (282 MHz, DMSO-d_6): δ -141.8 (br), -143.7 (s). ^{13}C NMR (101 MHz, DMSO-d_6) δ 181.39-178.65 (m, C=O), 98.98-96.24 (m, F-C), 54.81-48.28 (m. HOOC-C), 45.09- 43.31 (m, CH_2), 28.06-20.64, 19.74-17.70, 17.42-16.11 (m, CH_3). EA: C, 56.24%; H, 7.89%; F, 7.84%. GPC (DMF, PMMA calibration): M_n: 207 kDa, M_w: 323 kDa, PDI: 1.55. TGA: 388 °C (50% weight loss). DSC: T_g: 135 °C.

2-Fluoropropene – methacrylic acid copolymer 4e. This copolymer was prepared according to the procedure outlined in the synthesis of **4a** except that the amounts of Selectfluor, deionized water and aqueous $AgNO_3$ (0.1 M) solution were 5.0 mmol, 87 mL and 10 mL, respectively. Copolymer **4e** was thus obtained as a white solid. Yield: 560 mg (74%). FT-IR (KBr): v (cm^{-1}) 3425, 3167, 2995, 2947, 2598, 1704, 1474, 1454, 1387, 1364, 1270, 1184, 942, 835. ^{1}H NMR (400 MHz, DMSO-d_6): δ 12.41 (br s, COOH), 2.40-1.85, 1.85-1.56 (br, CH_2), 1.56-1.35, 1.33-1.19 (all br, CF-CH_3), 1.19-0.99, 0.99-0.83 (br, HOOCC-CH_3). ^{19}F NMR (282 MHz, DMSO-d_6): δ -141.7 (br), -143.3 (s). ^{13}C NMR (101 MHz, DMSO-d_6): δ 180.36-178.63 (m, C=O), 99.04-96.29 (m, F-C), 54.60-47.34 (HOOC-C), 45.04- 43.26 (m, CH_2), 27.43-20.63, 19.64-17.80, 17.23-16.10 (m, CH_3). EA: C, 56.50%; H, 7.80%; F, 9.89%. GPC (DMF, PMMA calibration): M_n: 188 kDa, M_w: 308 kDa, PDI: 1.63. TGA: 371 °C (50% weight loss). DSC: T_g: 122 °C.

2-Fluoropropene – methacrylic acid copolymer 4f. Selectfluor (2.13 g, 6.0 mmol), deionized water (85 mL) and sodium polymethacrylate (2.9 mL, M_n ~ 5400, M_w ~ 9500, 1.251 g/mL, 30 wt% in H_2O, equivalent to 10 mmol methacrylic acid monomer) were added successively into a three-necked flask at room temperature under nitrogen atmosphere. The mixture was stirred at room temperature for 10 min with the aid of a magnetic bar. Silver nitrate (1.2 mmol, 12.0 mL, 0.1 mol/L solution in water) was then added. The reaction mixture was kept from light and stirred at room temperature for 12 h. Hydrochloric acid (1 N) was then added dropwise until the pH was close to 3. The resulting mixture was filtered. The precipitate was washed with deionized water (3 × 20 mL) and dried in vacuo. The precipitate was then dissolved in acetone (50 mL)

and centrifuged. After filtration, The precipitate was dried in vacuo to give AgCl as a white solid. Yield: 138 mg (80% based on $AgNO_3$). The solution was concentrated to about 6 mL, to which water (100 mL) was added. The product copolymer precipitated out. The dissolution – precipitation procedure was repeated twice. Finally, the precipitate was collected and dried in vacuo to give copolymer **4f** as a white solid. Yield: 677 mg (91%). FT-IR (KBr): ν (cm^{-1}) 3443, 3149, 2993, 2945, 2610, 1704, 1473, 1454, 1387, 1364, 1187, 944, 836. ^1H NMR (400 MHz, DMSO-d_6): δ 12.46 (br s, COOH), 2.45-1.58 (br, CH_2), 1.56-1.34, 1.34-1.20 (all br, CF-CH_3), 1.20-1.03, 1.03-0.86 (br, HOOCC-CH_3). ^{19}F NMR (282 MHz, DMSO-d_6): δ -141.8 (br), -143.4 (s). ^{13}C NMR (101 MHz, DMSO-d_6): δ 180.36-178.63 (m, C=O), 98.60-96.27 (m, F-C), 54.58-47.68 (m, HOOC-C), 45.03- 43.26 (m, CH_2), 27.46-23.60, 22.71-20.88, 19.73-18.03 (m, CH_3). EA: C, 56.92%; H, 7.78%; F, 11.68%. GPC (DMF, PMMA calibration): M_n: 171 kDa, M_w: 293 kDa, PDI: 1.71. TGA: 361 °C (50% weight loss). DSC: T_g: 111 °C.

2-Fluoropropene – methacrylic acid copolymer 4g. This copolymer was prepared according to the procedure outlined in the synthesis of **4f** except that the amounts of Selectfluor, deionized water and aqueous $AgNO_3$ (0.1 M) solution were 7.0 mmol, 83 mL and 14 mL, respectively. Copolymer **4g** was thus obtained as a white solid. Yield: 621 mg (87%). FT-IR (KBr): ν (cm^{-1}) 3431, 3160, 2996, 2946, 2610, 1704, 1472, 1451, 1387, 1365, 1190, 943, 837. ^1H NMR (400 MHz, DMSO-d_6): δ 12.48 (br s, COOH), 2.44-1.59 (br, CH_2), 1.56-1.35, 1.34-1.20, 1.20-1.03, 1.03-0.89 (all br, CH_3). ^{19}F NMR (282 MHz, DMSO-d_6): δ -141.9 (br), -143.5 (s). ^{13}C NMR (101 MHz, DMSO-d_6): δ 180.23-178.56 (m, C=O), 98.60-96.27 (m, F-C), 54.55-47.98 (m, HOOC-C), 45.09-43.21 (m, CH_2), 27.22-23.81, 25.04-23.96, 22.53-20.83, 19.49-18.07 (m, CH_3). EA: C, 57.52%; H, 7.99%; F, 14.97%. GPC (DMF, PMMA calibration): M_n: 138 kDa, M_w: 261 kDa, PDI: 1.89. TGA: 361 °C (50% weight loss). DSC: T_g: 101 °C.

2-Fluoropropene – methacrylic acid copolymer 4h. This copolymer was prepared

according to the procedure outlined in the synthesis of **4f** except that the amounts of Selectfluor, deionized water and aqueous $AgNO_3$ (0.1 M) solution were 8.0 mmol, 81 mL and 16 mL, respectively. Copolymer **4h** was thus obtained as a white solid. Yield: 580 mg (83%). FT-IR (KBr): *v* (cm^{-1}) 3434, 2996, 2946, 2616, 1704, 1471, 1451, 1387, 1367, 1196, 944, 837. ^{1}H NMR (400 MHz, DMSO-d_6): δ 12.49 (br s, COOH), 2.51-1.59 (br, CH_2), 1.58-1.35, 1.34-1.20, 1.20-1.04, 1.04-0.89 (all br, CH_3). ^{19}F NMR (282 MHz, DMSO-d_6): δ -141.8 (br), -143.5 (s). ^{13}C NMR (101 MHz, DMSO-d_6): δ 180.19-178.57 (m, C=O), 98.54-96.25 (m, F-C), 54.10-47.23 (m, HOOC-C), 45.05-43.21 (m, CH_2), 27.46-23.83, 22.62-20.94, 19.68-18.20 (m, CH_3). EA: C, 57.83%; H, 7.74%; F, 17.36%. GPC (DMF, PMMA calibration): M_n: 115 kDa, M_w: 227 kDa, PDI: 1.96. TGA: 353 °C (50% weight loss). DSC: T_g: 90 °C.

2-Fluoropropene – methacrylic acid copolymer 4i. This copolymer was prepared according to the procedure outlined in the synthesis of **4f** except that the amounts of Selectfluor, deionized water and aqueous $AgNO_3$ (0.1 M) solution were 9.0 mmol, 79 mL and 18 mL, respectively. Copolymer **4i** was thus obtained as a white solid. Yield: 633 mg (93%). FT-IR (KBr): *v* (cm^{-1}) 3434, 2999, 2946, 2604, 1704, 1471, 1457, 1387, 1367, 1199, 944, 837. ^{1}H NMR (400 MHz, DMSO-d_6): δ 12.47 (br s, COOH), 2.57-1.62 (br, CH_2), 1.57-1.37, 1.34-1.20, 1.20-1.04, 1.04-0.89 (all br, CH_3). ^{19}F NMR (282 MHz, DMSO-d_6): δ -141.9 (s), -142.4 (s), -143.4 (s). ^{13}C NMR (101 MHz, DMSO-d_6): δ 179.68-178.63 (m, C=O), 98.57-96.29 (m, F-C), 53.33-47.50 (m, HOOC-C), 44.91-43.33 (m, CH_2), 27.26-23.77, 22.48-21.02, 19.59-18.33 (m, CH_3). EA: C, 58.05%; H, 7.80%; F, 19.51%. GPC (DMF, PMMA calibration): M_n: 76 kDa, M_w: 213 kDa, PDI: 2.80. TGA: 343 °C (50% weight loss). DSC: T_g: 80 °C.

Supplementary Tables

Supplementary Table 1. Calculated Molar Contents of Vinyl Fluoride in Copolymers 2

**2**	Selectfluor (mol %)	Elemental analysis[a]		C/F	Calculated VF content in **2** (x mol %)		
		C (%)	F (%)		Avg.[b]	Max.[c]	Min.[d]
**2a**	10	48.06	1.77	27.15	6.8	8.8	4.9
**2b**	20	46.31	4.15	11.16	16.1	18.0	14.1
**2c**	30	47.42	6.81	6.96	25.0	26.8	23.1
**2d**	40	48.32	9.43	5.12	32.9	34.7	31.2
**2e**	50	47.47	11.94	3.98	41.1	42.9	39.4
**2f**	60	49.40	15.05	3.28	48.4	50.1	46.8
**2g**	70	48.93	16.84	2.91	53.6	55.3	51.9
**2h**	80	49.13	18.86	2.61	58.6	60.2	57.0
**2i**	90	49.27	21.82	2.26	65.6	76.2	64.0

[a] The systematic deviation is ±0.4% for carbon and ±0.5% for fluorine. [b] Calculated based on C/F ratio. [c] Calculated based on (C% – 0.4%)/(F% + 0.5%). [d] Calculated based on (C% + 0.4%)/(F% – 0.5%).

Supplementary Table 2. Calculated Molar Contents of 2-Fluoropropene in Copolymers 4

**4**	Selectfluor (mol %)	Elemental analysis[a]		C/F	Calculated 2-FP content in **4** (x mol %)		
		C (%)	F (%)		Avg.[b]	Max.[c]	Min.[d]
**4a**	10	50.74	0.77	65.90	3.80	6.27	1.33
**4b**	20	51.88	2.84	18.27	13.4	15.7	11.0
**4c**	30	52.07	5.05	10.31	23.1	25.4	20.8
**4d**	40	56.24	7.84	7.173	32.4	34.5	30.3
**4e**	50	56.50	9.89	5.713	39.8	41.9	37.8
**4f**	60	56.92	11.68	4.873	45.9	47.9	43.9
**4g**	70	57.52	14.97	3.842	56.5	58.4	54.5
**4h**	80	57.83	17.36	3.331	63.7	65.7	61.8
**4i**	90	58.05	19.51	2.975	70.0	71.9	68.2

[a] The systematic deviation is ±0.4% for carbon and ±0.5% for fluorine. [b] Calculated based on C/F ratio. [c] Calculated based on (C% – 0.4%)/(F% + 0.5%). [d] Calculated based on (C% + 0.4%)/(F% – 0.5%).

Supplementary Figures

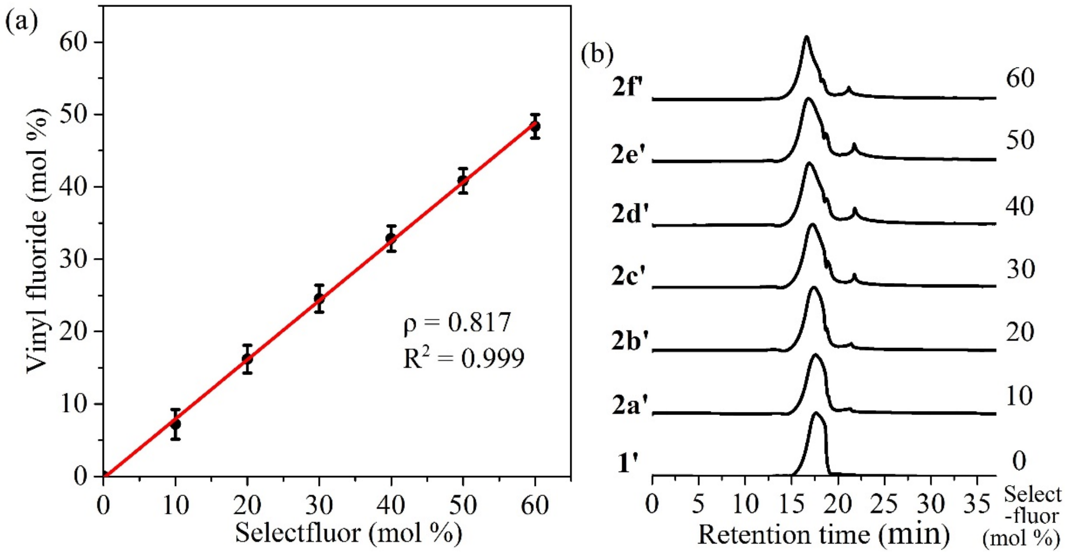

Supplementary Figure 1. Controlled decarboxylative fluorination of 1' in water. Poly(acrylic acid, sodium salt) (average M_w ~ 15,000, 35 wt% in H_2O, Aldrich) was used. $[Selectfluor]_0/[AgNO_3]_0$ = 5:1. (a) Dependence of the molar percentage of vinyl fluoride in copolymers **2'** on the amount of Selectfluor. (b) GPC (H_2O) chromatograms of copolymers **2a' – 2f'** and PAA **1'** (all in the form of sodium salt).

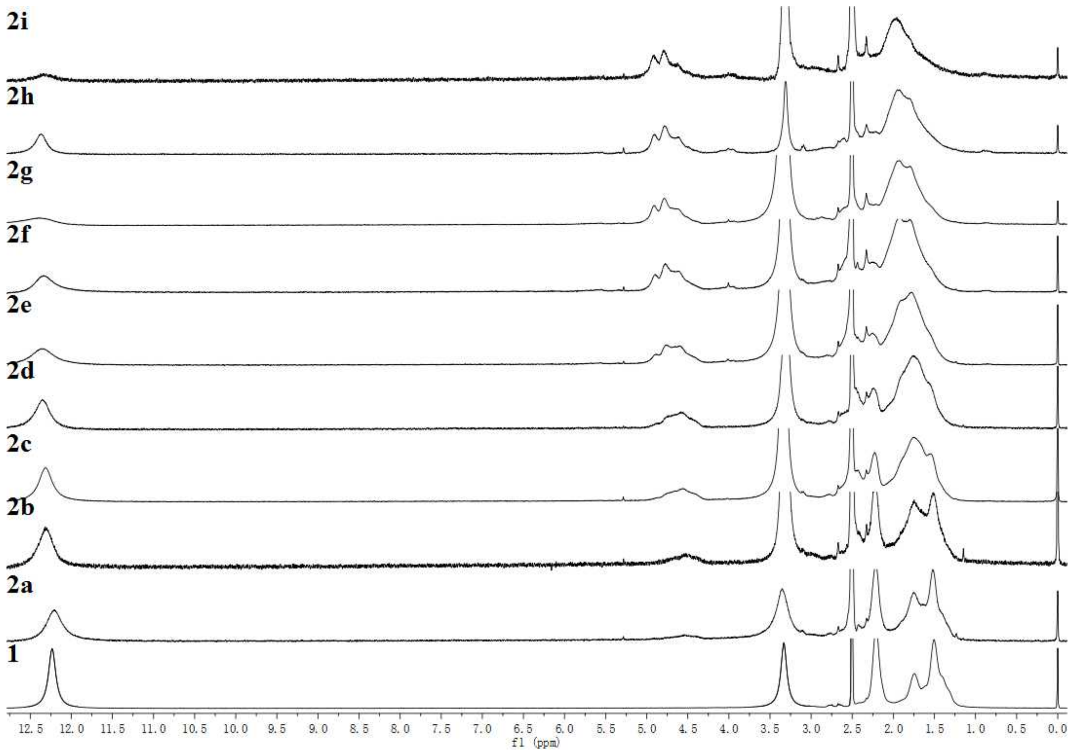

Supplementary Figure 2. ^{1}H NMR (DMSO-d_6) spectra of 1 and 2a–2i.

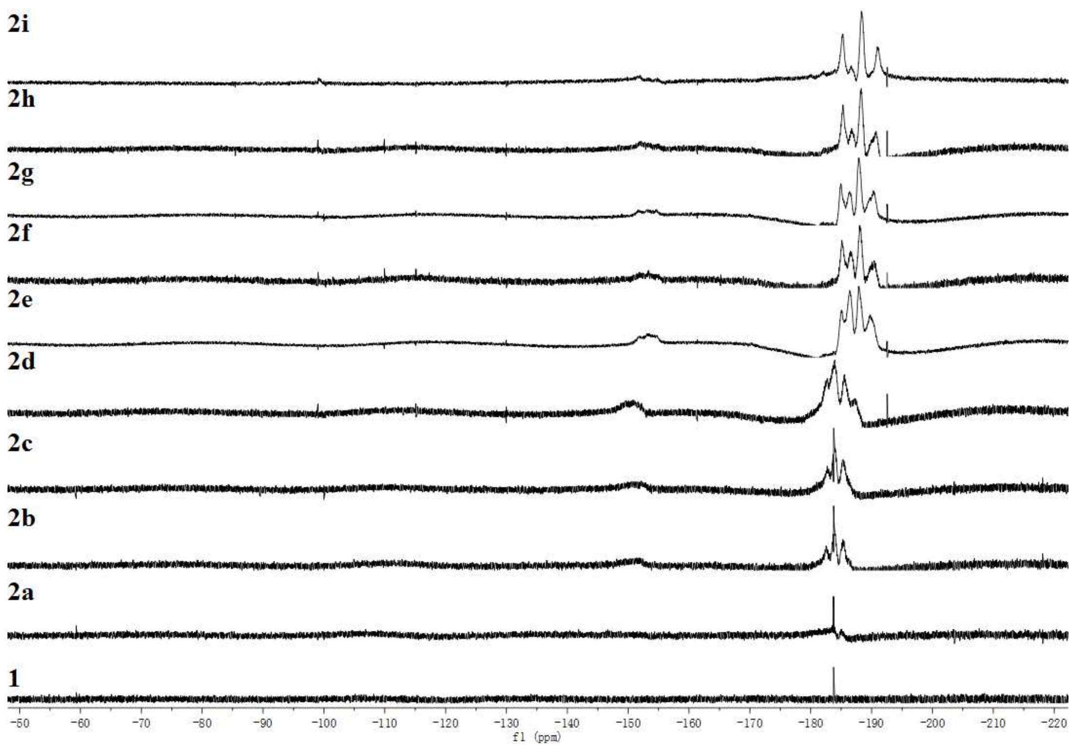

Supplementary Figure 3. ^{19}F NMR (DMF) spectra of 1 and copolymers 2a–2i.

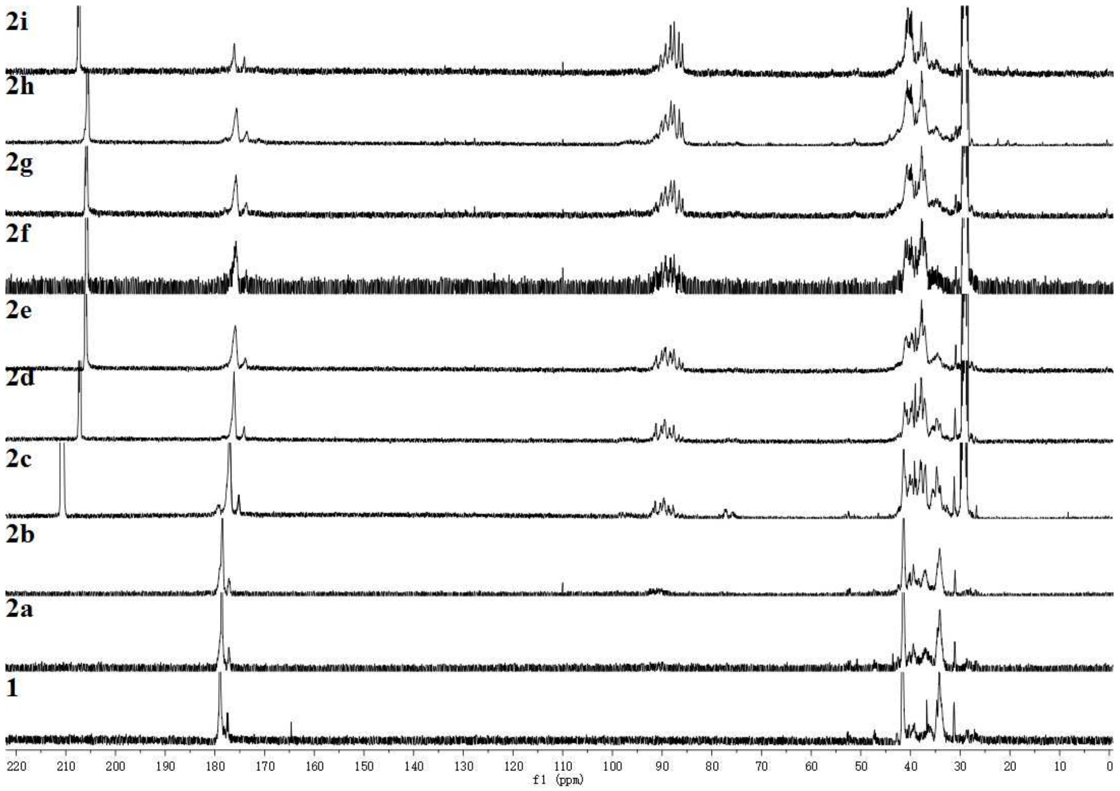

Supplementary Figure 4. ^{13}C NMR spectra of PAA 1 and copolymers 2a–2i.

(Solvent: D_2O for **1**, **2a** and **2b**, D_2O/acetone-d_6 for **2c**, and acetone-d_6 for **2d–2i**.)

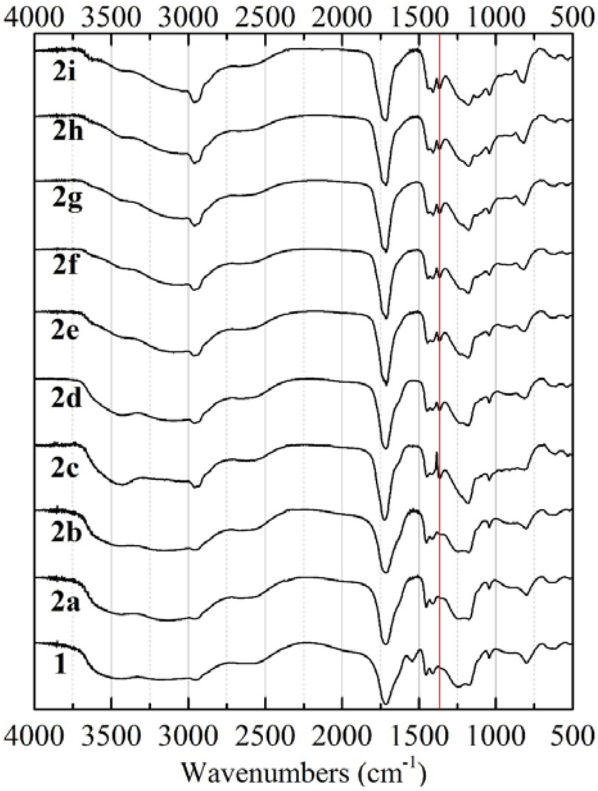

Supplementary Figure 5. FI-IR (KBr) spectra of PAA 1 and copolymers 2a–2i.

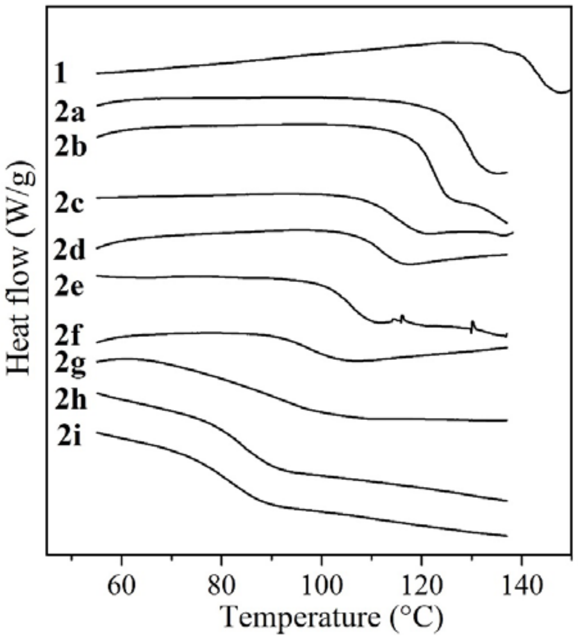

Supplementary Figure 6. DSC thermograms of PAA 1 and copolymers 2a–2i.

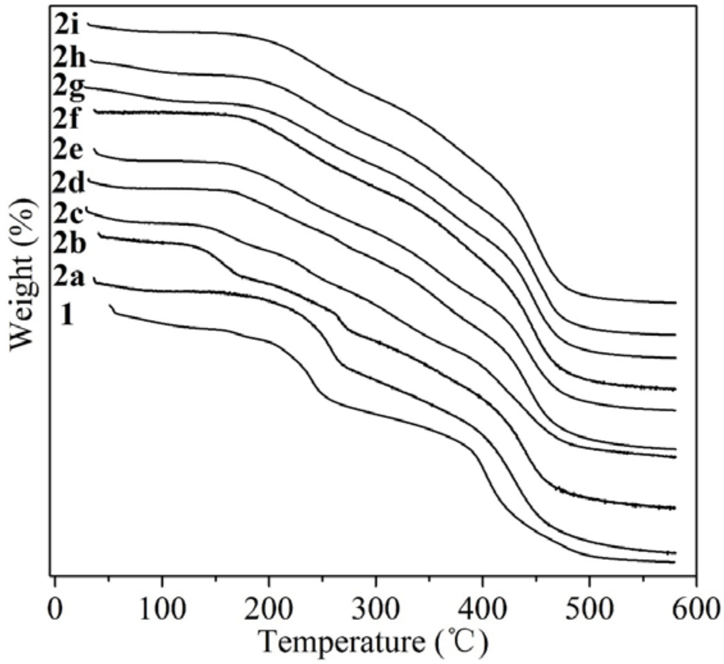

Supplementary Figure 7. TGA plot of PAA 1 and copolymers 2a–2i.

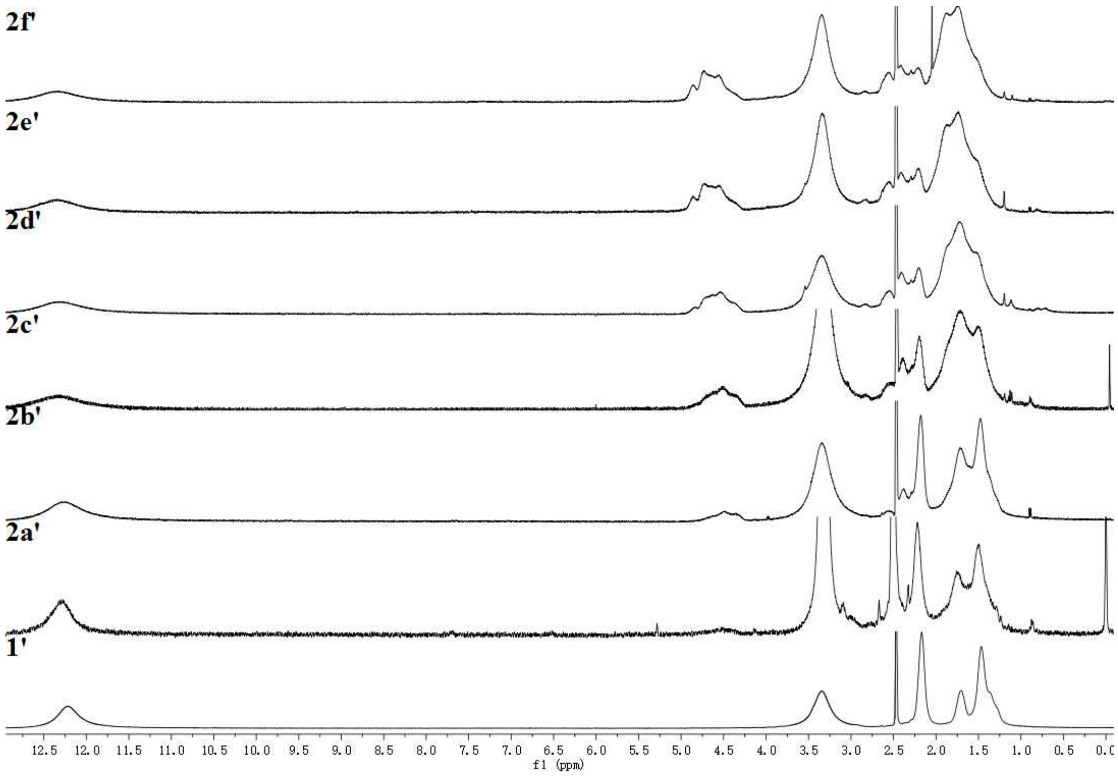

Supplementary Figure 8. ^{1}H NMR (DMSO-d_6) spectra of 1' and 2a'–2f'.

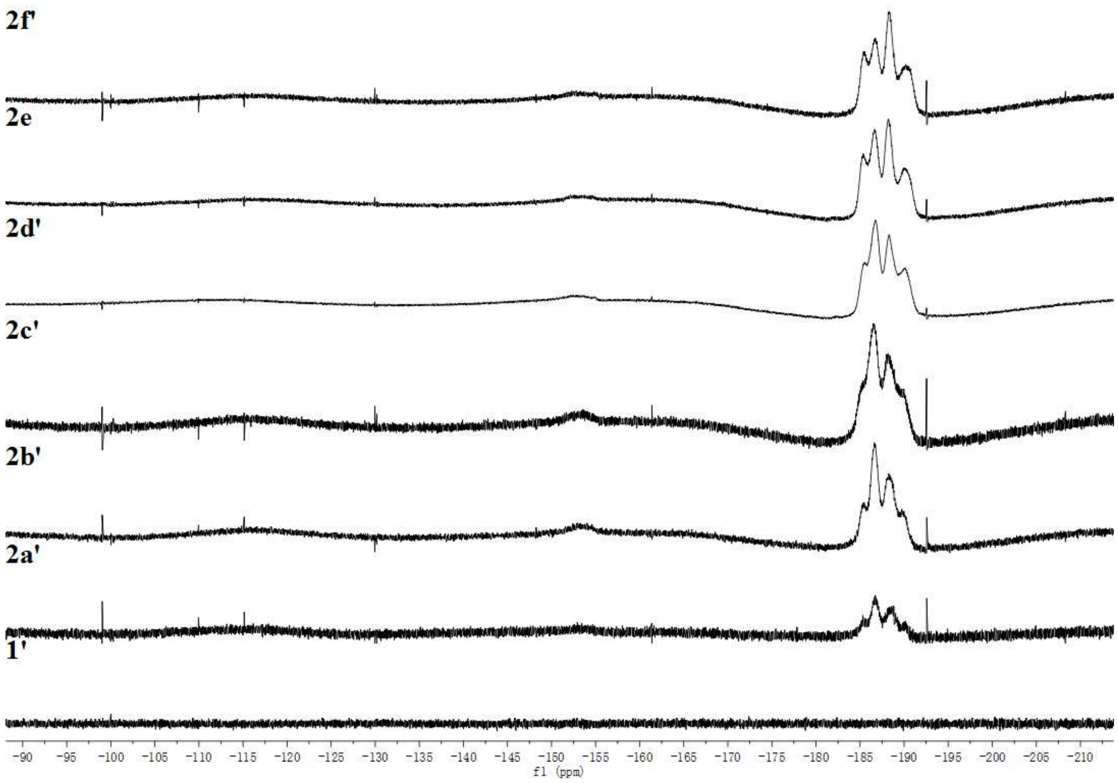

Supplementary Figure 9. ^{19}F NMR (DMF) spectra of 1' and copolymers 2a'–2f'.

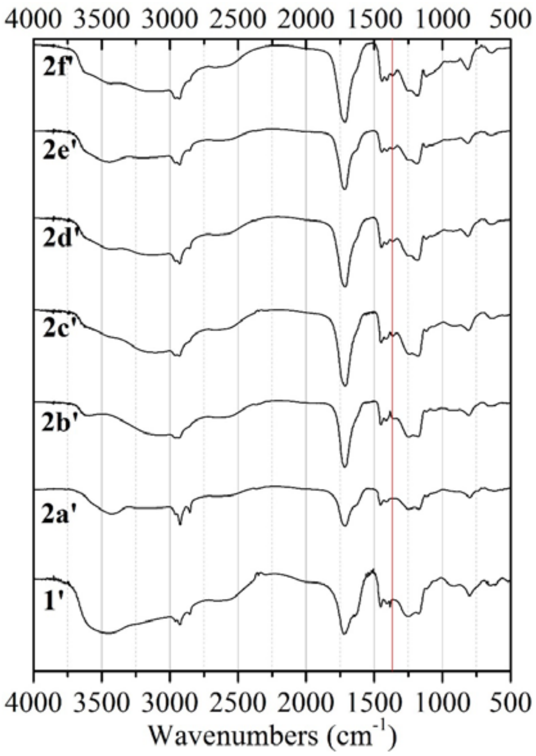

Supplementary Figure 10. FT-IR (KBr) spectra of 1' and copolymers 2a'–2f'.

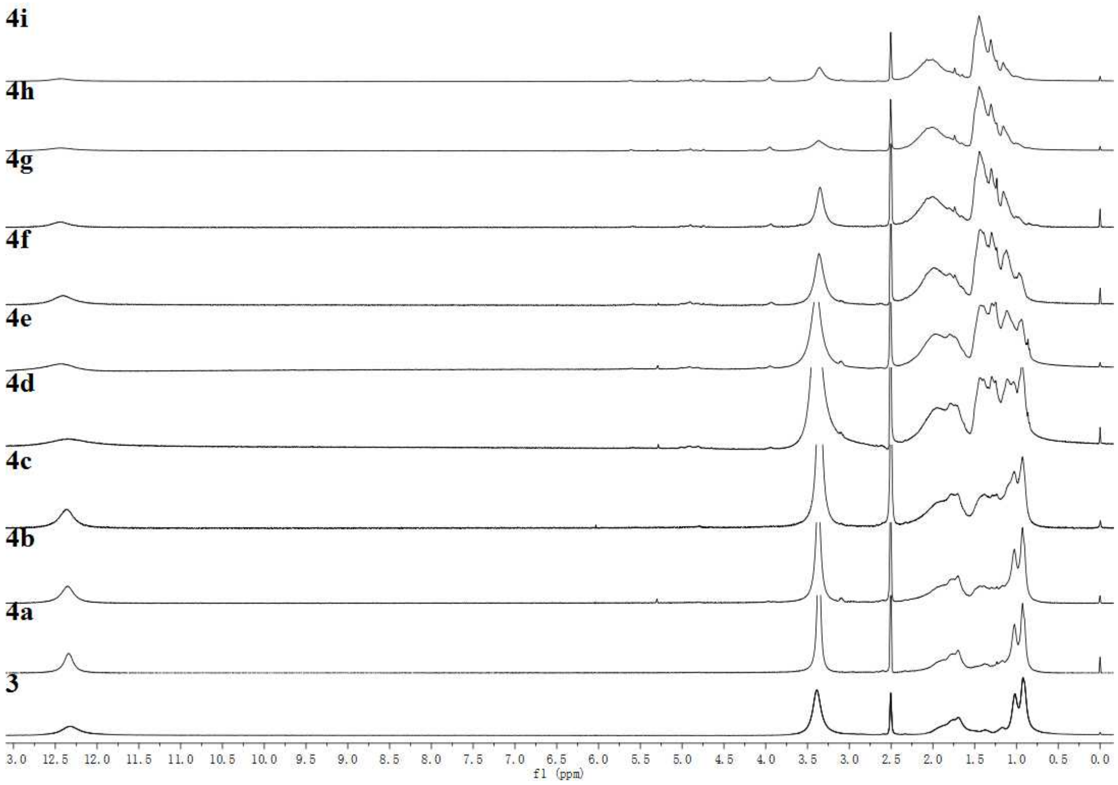

Supplementary Figure 11. ^{1}H NMR (DMSO-d$_6$) spectra of 3 and 4a–4i.

(For **3**, **4a** and **4b**, a small amount of D_2O was used as the cosolvent to improve the solubility.)

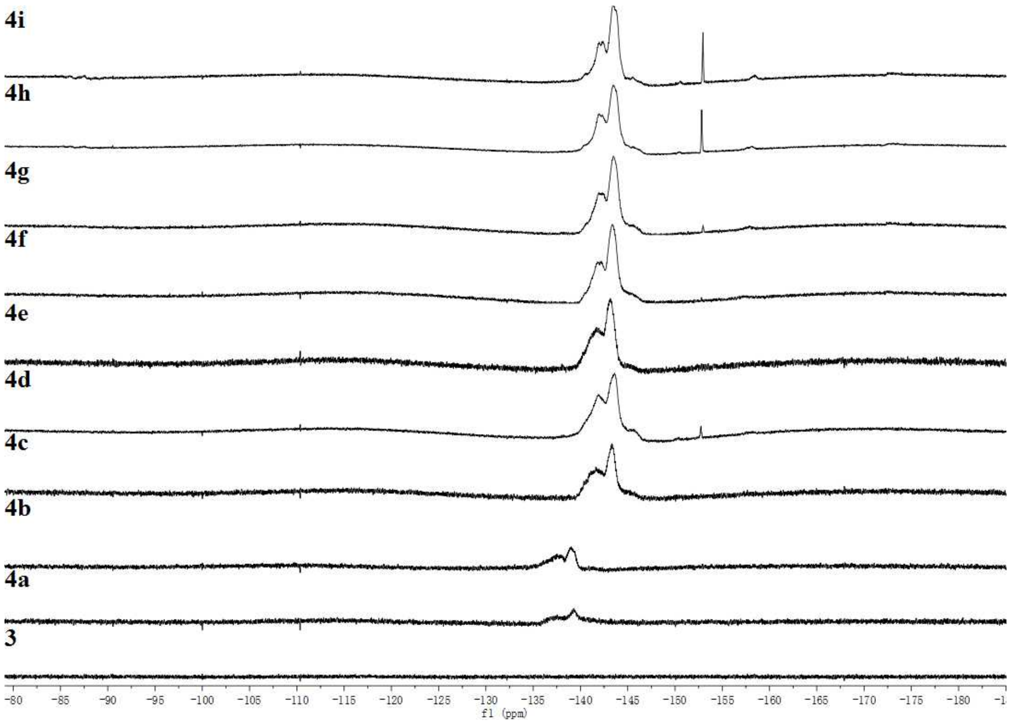

Supplementary Figure 12. ^{19}F NMR (DMSO-d$_6$) spectra of 3 and 4a–4i.

(For **3**, **4a** and **4b**, a small amount of D_2O was used as the cosolvent to improve the solubility.)

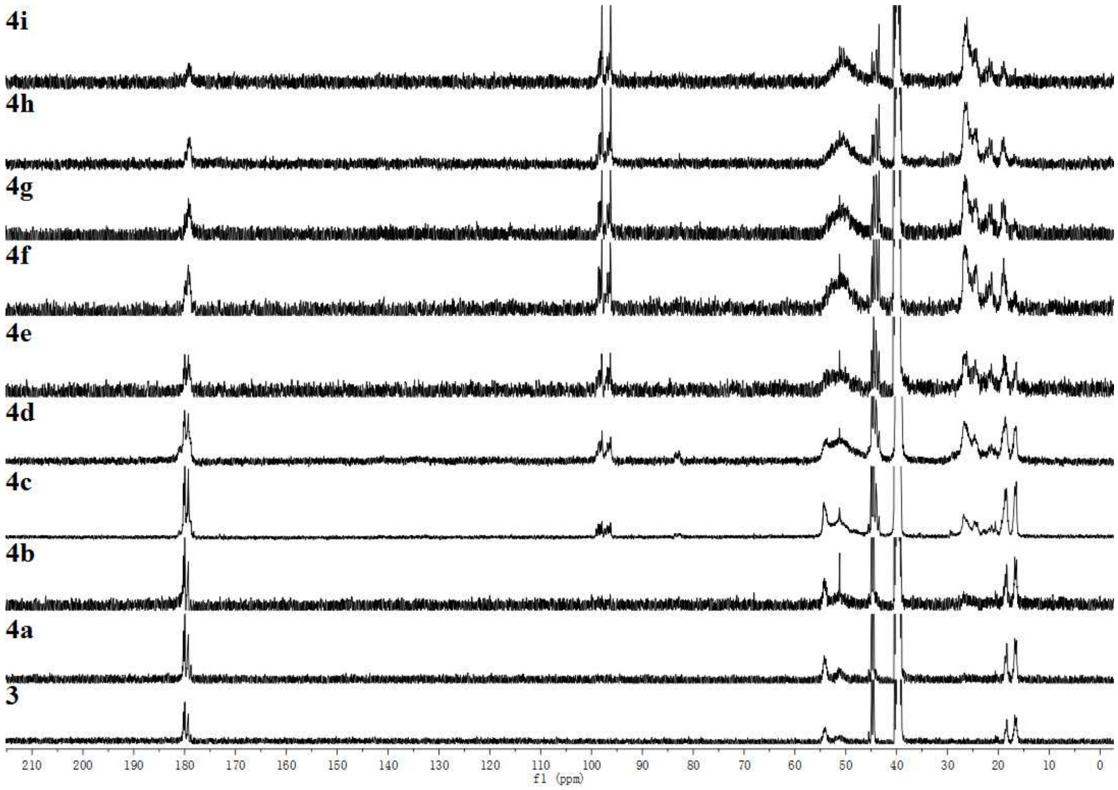

Supplementary Figure 13. ^{13}C NMR (DMSO-d_6) spectra of 3 and 4a–4i.

(For **3**, **4a** and **4b**, a small amount of D_2O was used as the cosolvent to improve the solubility.)

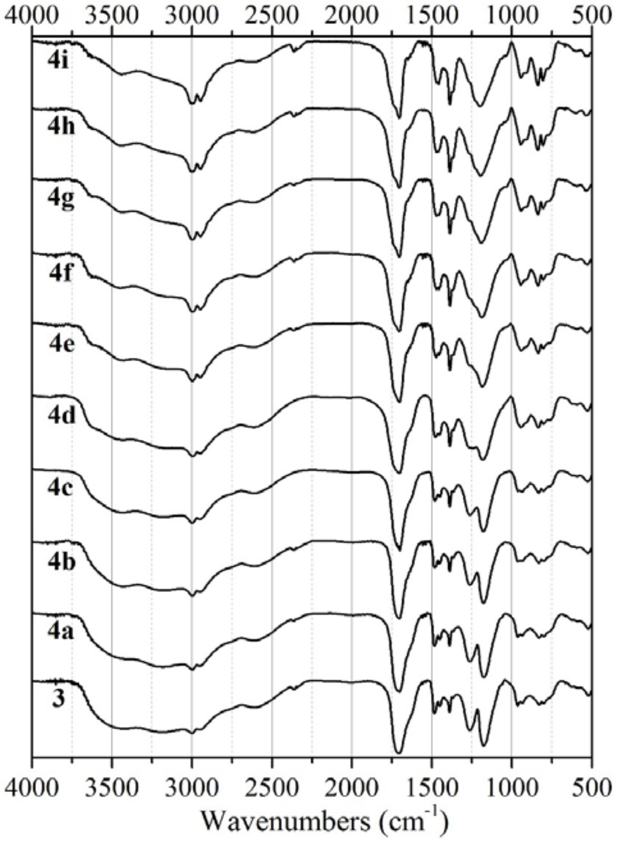

Supplementary Figure 14. FT-IR (KBr) spectra of 3 and copolymers 4a–4i.

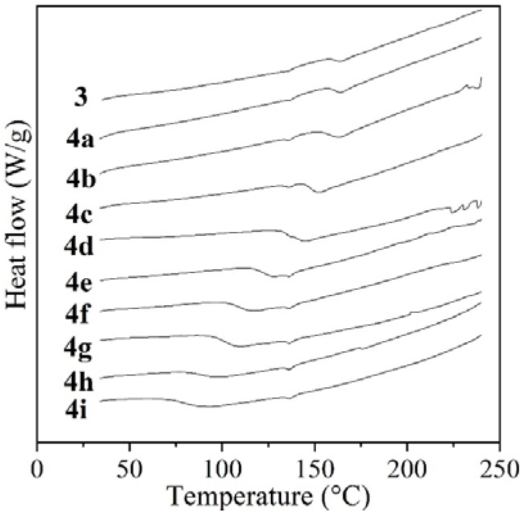

Supplementary Figure 15. DSC thermograms of PMAA 3 and copolymers 4a–4i.

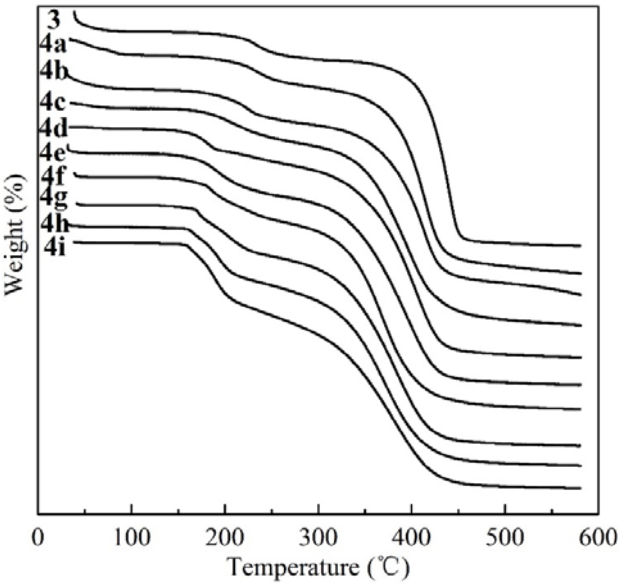

Supplementary Figure 16. TGA plot of PMAA 3 and copolymers 4a–4i.
